# Localized Nanoscale Formation of Vanadyl Porphyrin 2D MOF Nanosheets and Their Optimal Coupling to Lumped Element Superconducting Resonators

**DOI:** 10.1021/acs.jpcc.4c07265

**Published:** 2024-12-25

**Authors:** Ignacio Gimeno, Fernando Luis, Carlos Marcuello, Maria Carmen Pallarés, Anabel Lostao, Marina Calero de Ory, Alicia Gomez, Daniel Granados, Inés Tejedor, Eva Natividad, Ainhoa Urtizberea, Olivier Roubeau

**Affiliations:** † Instituto de Nanociencia y Materiales de Aragón (INMA), CSIC − Universidad de Zaragoza, Plaza San Francisco s/n, Zaragoza 50009, Spain; ‡ Laboratorio de Microscopias Avanzadas (LMA), Universidad de Zaragoza, Ed. I+D+i. Mariano Esquillor s/n, Zaragoza 50018, Spain; § Fundación ARAID, Av. Ranillas 1-D, Zaragoza 50018, Spain; ∥ Centro de Astrobiología, CSIC − INTA, Torrejón de Ardoz, Madrid 28850, Spain; ⊥ IMDEA Nanociencia, Cantoblanco, Madrid 28049, Spain; # Instituto de Nanociencia y Materiales de Aragón (INMA), CSIC − Universidad de Zaragoza, Campus Rio Ebro, María de luna 3, Zaragoza 50018, Spain

## Abstract

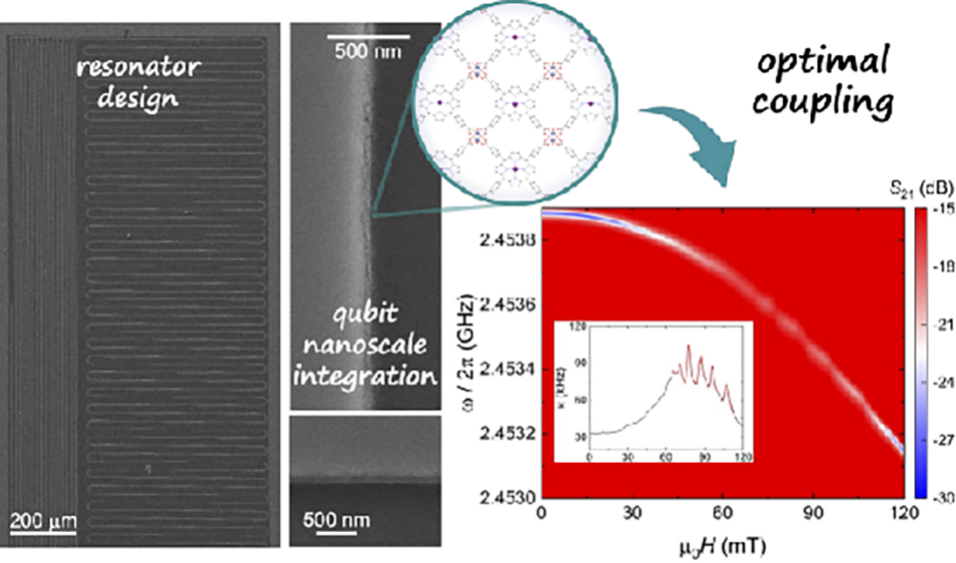

A strategy toward the realization of a quantum spin processor involves the coupling of spin qubits and qudits to photons within superconducting resonators. To enable the realization of such hybrid architecture, here we first explore the design of a chip with multiple lumped-element LC superconducting resonators optimized for their coupling to distinct transitions of a vanadyl porphyrin electronuclear qudit. The controlled integration of the vanadyl qudit onto the superconducting device, both in terms of number and orientation, is then attained using the *in situ* formation of nanosheets of a 2D framework built on the vanadyl qudit as a node. Low-temperature transmission experiments demonstrate the coupling of photons in resonators with different frequencies to the targeted electronuclear transitions of the vanadyl qudit, also confirming the control over the vanadyl qudit node orientation. The derived collective spin-photon couplings in the 0.3–1.6 MHz range then allow to estimate enhanced, optimal, single spin photon couplings up to 4 Hz.

## Introduction

On the way toward the development of useful quantum computers, the electronic spin carried by paramagnetic molecules, either organic radicals or transition metal ion complexes, has arisen as a valid quantum hardware alternative.^1−5^ A key advance in this field was the demonstration that molecular spin qubits can exhibit sufficiently long quantum coherence times,^6,7^ thereby opening the way to take advantage of the fact that a single synthesis results in molar numbers of perfectly identical qubits. Chemical design can also provide the conditions to operate quantum gates or even algorithms at the single-molecule level, either through weakly interacting multiple qubits or thanks to the coupling with nuclear spins.^8−13^ Nevertheless, a crucial challenge to develop quantum processors based on molecular spins is their individual reading and manipulation, in large numbers. For this, an approach inspired by circuit Quantum Electrodynamics (cQED)^14^ involves their integration at the surface of superconducting circuits to build a hybrid quantum architecture.^15−17^ The ability to manipulate isolated molecules through solution-based methods is clearly a facilitating aspect for this, although still challenging. Aspects related with maintaining molecular properties once integrated may also prove problematic. For these reasons, state-of-the-art experiments have so far been done mainly on large spin ensembles in macroscopic crystals.^18−24^ Moving toward the manipulation and readout of single spins with this technology first relies on improving the sensitivity of superconducting resonators, which is limited by the typically very weak spin-photon interaction. In order to overcome this limitation, nanoconstrictions can be fabricated to concentrate and locally enhance the photon magnetic field by several orders of magnitude.^25^ However, the precise molecular location with respect to the chip surface becomes then critical. In order to optimize the spin-photon coupling, molecular qubits must be disposed at specific locations of the superconducting surface, and with a controlled orientation.

Recently, nanodroplets of the organic radical 2,2-diphenyl-1-picrylhydrazyl (DPPH) were deposited onto coplanar superconducting resonators,^26^ by Dip-Pen nanolithography (DPN), which is based on atomic force microscopy (AFM).^27^ The results show that a quite strong coupling of the molecular qubits to the circuit photon modes is attainable. DPN has also allowed to dispose Y hydroxycarbonate nanoparticles doped with an adjustable number of Gd(III) spin qubits.^28^ We have also reported that diluted nanodomains of 2D frameworks with metalloporphyrin qubit nodes [{MTCPP}Zn_2_(H_2_O)_2_] (H_2_TCPP is 5,10,15,20-tetracarboxyphenylporphyrin) can be transferred through the Langmuir–Schaefer (LS) technique onto different substrates or directly formed *in situ* on the surface of superconducting lines.^29,30^ This strategy has the advantage of forcing an homogeneous and controlled orientation of the qubit nodes, through the underlying periodicity of the 2D framework. In addition, broad-band spectroscopy experiments performed on [VOTCPPEt] crystals (H_2_TCPPEt is the ethyl ester of 5,10,15,20-tetracarboxyphenylporphyrin) have shown that the vanadyl porphyrin moiety fulfills the conditions to act as a universal 4-qubit processor or, equivalently, as a d = 16 qudit.^19^

**Scheme 1:**
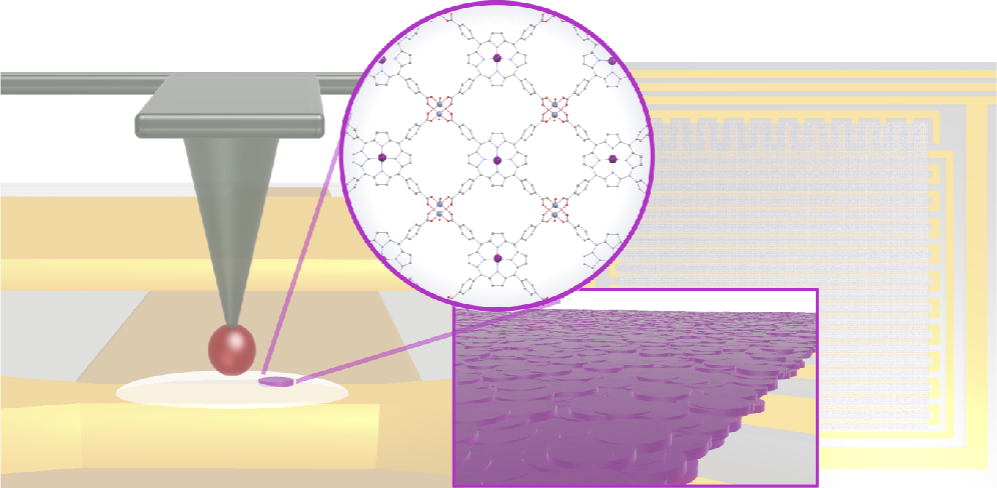
Scheme of *In-Situ* Formation of Isolated Nanodomains of the [{VOTCPP}Zn_2_(H_2_O)_2_] 2D MOF on a Circuit Nanoconstriction (left) and Coverage of a Lumped Element Resonator by an Ultrathin Film of MOF Nanodomains (right)

Here, we take a step forward, by designing superconducting lumped-element resonators (LERs) specifically tuned to match the frequencies of certain electronuclear spin transitions from a vanadyl porphyrin qudit node.^19,30^ First, the conditions to form *in situ* nanodomains of the 2D [{VOTCPP}M_2_(H_2_O)_2_] MOFs are explored. Then, a chip with various LERs is covered with 4 layers of ultrathin films of [{VOTCPP}Zn_2_(H_2_O)_2_] (Scheme 1), thereby adjusting the number of spins to the LERs expected sensitivity. Microwave transmission experiments show relatively strong couplings to the target spin transitions, thereby enabling the manipulation of the vanadyl electronuclear qudit within this hybrid scheme.

## Methods

### Lumped Element Resonators Fabrication

The nanofabrication of the superconducting niobium resonators was conducted at the Center for Nanofabrication NanoFabLab at IMDEA Nanoscience. The process starts with the pretreatment of a 275 μm thick silicon (Si) substrate in a 1% hydrofluoric acid bath to remove the native silicon oxide layer. A 100 nm thick niobium (Nb) film is then deposited onto the Si substrate using confocal DC AJA magnetron sputtering, with a chamber base pressure below 2 × 10^–8^ Torr. The Nb deposition is carried out at 100 W, with an argon (Ar) pressure of 1.5 mTorr and a flow rate of 15 sccm.

Afterward, maskless laser writer lithography is employed to pattern the chip design onto the Nb film. A layer of negative photoresist (AZ2070) is spin-coated onto the surface and exposed to a 405 nm laser at room temperature. Following the development of the resist, reactive ion etching is performed using a gas mixture of argon (10 sccm, 0.1 mTorr) and sulfur hexafluoride (SF_6_) (20 sccm, 10 mTorr). The process concludes with the removal of the remaining photoresist by boiling in acetone, followed by an isopropanol rinse.

### Transfer of Langmuir–Schaefer Film of [{VOTCPP}Zn_2_(H_2_O)_2_] Nanodomains

This was done as previously described for Si, Mylar and quartz substrates,^30^ using the chip with LERs as substrate. For this, a KSV-NIMA trough model KN 2003, with dimensions of 580 mm × 145 mm, housed in a clean room inside a closed cabinet and whose temperature is maintained at 293 ± 1 K, was used (INMA, CSIC and Universidad de Zaragoza). The trough was carefully cleaned with acetone and chloroform, filled and emptied twice with Milli-Q water (ρ = 18.2 MΩ·cm), and ultimately filled with a 0.1 M ZnCl_2_ solution made with Milli-Q water and filtered prior to use on a 0.2 μm membrane to remove the small amounts of Zn oxychloride formed upon solubilization. The subphase was then carefully cleaned by closing the barriers down to 40 mm distance and mild surface-touch vacuuming intrabarriers area. After opening the barriers to the maximum area, the system was let to equilibrate for 5 min, and a [VOTCPP] CHCl_3_:CH_3_OH solution (3:1 v:v) was carefully spread drop-by-drop onto the subphase using a Hamilton microsyringe held very close to the subphase surface. After 20 min letting evaporate the organic solvents, compression was performed at constant speed of 7.5 cm^2^·min^–1^ until reaching a surface pressure of 5 mN/m. Four successive transfers of the [{VOTCPP}Zn_2_(H_2_O)_2_] nanodomains film formed at the air–water interface were then carried out by horizontal-dipping at a surface pressure of 5 mN·m^–1^, the substrate being approached to the surface at 0.2 mm·min^–1^ and raised at 10 mm·min^–1^. Between successive transfers, the substrate was cleaned by gently flushing with Milli-Q water, submerging in Milli-Q water for 3 min, and drying under a N_2_ flush.

### Microwave Transmission Experiments

These were performed by mounting the chip with LERs covered by the 4 LS ultrathin film in a BlueFors LD450 dilution refrigerator equipped with a 1 T superconducting magnet (INMA, CSIC and Universidad de Zaragoza), and connecting it through coaxial cables to a Vector Network Analyzer with a measurement frequency bandwidth ranging from 100 MHz to 14 GHz. Device characterization is then done by measuring the transmitted signal *S*_21_.

## Results and Discussion

### Design of LERs

**Figure 1:**
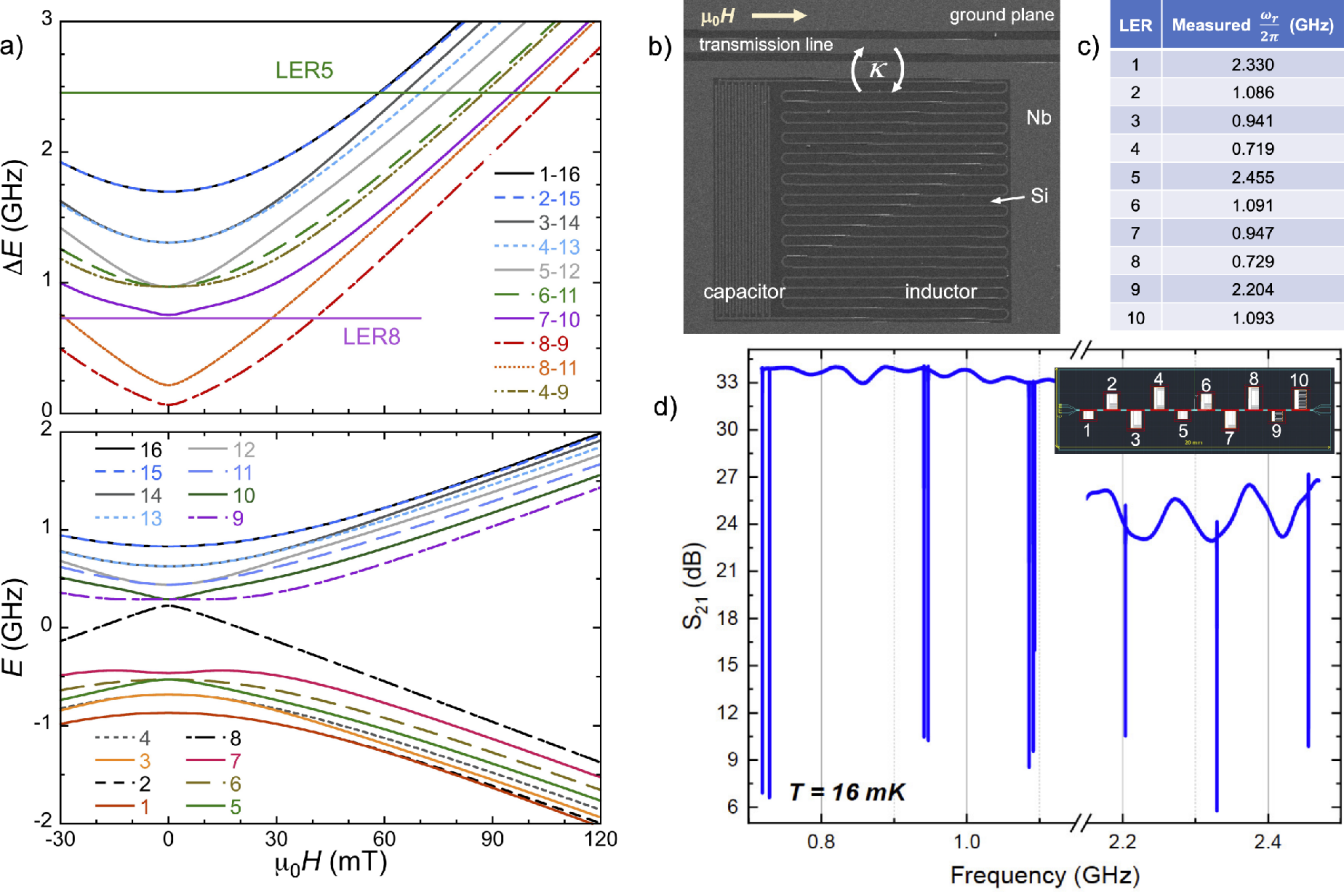
(a) Spin energy levels (bottom) and selected spin transition frequencies (top) associated with each [VOTCPP] node as calculated with Easyspin^39^ for *g*_*z*_ = 1.963, *g*_*x,*_*_y_* = 1.99, *A*_*z*_ = 475 MHz, *A*_*x,*_*_y_* = 172 MHz, and a magnetic field  applied along the *x* molecular axis. The horizontal lines in the top panel indicate the frequencies of the two LERs for which transmission measurements are reported in this work. Because the electronic and nuclear spins become entangled at low fields, each level is assigned an index n that follows the order of increasing energy. (b) Scanning electron microscopy image of LER5 showing the LC inductor–capacitor superconducting circuit coupled inductively to the chip common transmission line, which is parallel to the external magnetic field. (c) Measured resonance frequencies of the 10 LERs. (d) Microwave transmission amplitude vs frequency measured at *T* = 16 mK, showing sharp transmission dips near the resonances of the 10 LERs and therefore allowing to determine *ω*_*r*_.

Superconducting microwave resonators have been involved in the development of quantum computing architectures based on cQED.^31,32^ Among various materials, niobium has been used to develop high quality resonators for quantum computing applications,^17,20^ since its relatively high critical temperature (*T*_C_ ∼ 9 K) and large critical field can allow better stability against temperature and magnetic field fluctuations. LERs consist of series inductor–capacitor circuits acting as highly efficient microwave cavities coupled to a single transmission line. Their design allows frequency multiplexing and tuneability, opening the possibility of simultaneous characterization of different samples in a single chip.^33−35^ Each LER resonance frequency *ω*_*r*_ is defined by the inductor–capacitor geometry, . Here a device with ten Nb superconducting LERs coupled in parallel to a common coplanar waveguide transmission line (CPW) was designed so as to sense the different electronuclear spin transitions of the [VOTCPP] node in the 2D [{VOTCPP}M_2_(H_2_O)_2_] MOF nanodomains (Figure 1a).^36^ The electronuclear spin states arising from the coupled *S* = 1/2 and *I* = 7/2 electronic and nuclear spins provide the basis to encode a *d* = 16 qudit.^19^ It is therefore key to experimentally allow the reading or operation of specific resonant transitions. Thus, seven LERs were designed to have , slightly above the level anticrossings of the electronuclear clock-like transitions in the low magnetic field regime,^37^ whereas the three remaining LERs, with frequencies in the range 2.1–2.5 GHz, were aimed at the efficient coupling to spin transitions in the intermediate field range (Figures 1, S1).

The cryogenic characterization of the chip was performed in a cryogen-free dilution refrigerator equipped with microwave coaxial lines and measuring the transmission *S*_21_ as a function of frequency with a network vector analyzer. Figure 1d shows the *S*_21_ spectrum obtained at *T* = 16 mK by applying a driving power of −65 dBm to the readout transmission line. Each transmission dip corresponds to the resonance of a LER. Fitting each of them allows to derive the relevant resonator parameters,^38^ i.e., *ω*_*r*_ and the quality factors, *Q*_*i*_ and *Q*_*c*_, associated with the intrinsic resonator losses and to its coupling to the readout line, respectively. These values match those obtained from the simulated designs in a very consistent manner, indicating that the fabrication process is reliable. We find *Q*_*c*_ values in the range 60–105 × 10^3^, while *Q*_*i*_ values are 0.8 up to 2 × 10^6^, which result in photon life times longer than 40 μs. These figures validate their interest to reach a significant coupling of resonant photons to molecular spins.

### In-Situ Formation of 2D MOF Nanodomains

Even though the designed LERs provide an ideal platform to efficiently couple microwave photons to molecular spin qubits, the way these are integrated onto the superconducting circuits remains a key challenge. Indeed, any gap between the resonator surface and the integrated material can waste a significant fraction of the resonator mode volume, typically the first few microns atop the circuit surface, where the electromagnetic field is most intense. This is the case when macroscopic crystals are used, since any imperfection of the crystal face in contact with the circuit can result in separations of the order of few to tens of microns.^20,24^ An alternative is to deposit molecules directly from solution. Nevertheless the resulting random molecular orientation introduces a huge line broadening unless the molecular spins are close to isotropic, which is only the case of some organic free radicals.^23,26^ The integration of nanodomains of 2D MOFs allows to optimize chip-sample interface while still ensuring a quite homogeneous orientation of its qubit nodes.^29^ A way to further enhance the spin-photon coupling, is to fabricate constrictions at the superconducting line that defines the LER’s inductor. Molecules then need to be integrated at a distance from the constriction that is smaller than the constriction width.^26^

We therefore design here a DPN-based lithography adaptation of the protocol previously used to cover the surface of a Nb CPW with nanodomains of [{VOTCPP}Zn_2_(H_2_O)_2_],^30^ with the aim of better controlling their location, and possibly their size. This new protocol mimics *in situ* the conditions under which MOF nanodomains are formed at the air–water interface of a Langmuir trough, by depositing μ-droplets of a MeOH:CHCl_3_ solution of [VOTCPP] onto an aqueous ZnCl_2_ droplet previously deposited over the Nb substrate.

Thus, we have used the AFM tip of a DPN setup to first leave on Si substrates a drop of an aqueous MCl_2_ ink (M = Cu, 1 mM, or M = Zn, 100 mM), and then atop drop(s) of MeOH:CHCl_3_ [VOTCPP] ink solutions. Isolated nanodomains do seem to form *in situ* (Figure S2), but unfortunately their locations cannot be controlled with the required, nanoscopic accuracy (see Supporting Information for details). Most likely, this is due to the spreading of the subjacent aqueous nanodroplet over large areas of the flat native SiO_2_ layer that covers the Si substrate. Since this is intrinsic to the contact angle of the aqueous droplet, similar results can be expected on Nb superconducting circuits, due to the existence of a similar Nb_2_O_5_ native layer (see below).

In order to circumvent this problem, the same procedure was repeated within the physically confined area of μ-wells, typically ca. 100 μm wide and 100 nm deep, fabricated in a Si wafer surface by focused-ion beam (FIB) lithography (Figure S3). AFM observations and Raman spectroscopy, together with negative controls, allowed to confirm that [{VOTCPP}M_2_(H_2_O)_2_] (M = Zn, Cu) nanodomains are indeed formed, and cover entirely the surface of the μ-wells (see Figures S4–S7). These results convincingly show that nanodomains of the [{VOTCPP}M_2_(H_2_O)_2_] (M = Zn, Cu) MOFs can be formed *in situ* through DPN under suitable conditions. However, achieving a nanoscopic control over the position of such deposits does not seem to be possible without an artificial confinement, at least on native oxide surfaces. In the following, we therefore turned to using the LS technique, as described before, to fully cover the sensitive region of LERs, instead of aiming at disposing few isolated nanodomains on constrictions.

### Langmuir–Schaefer Film on LERs

The film was formed and transferred under similar conditions as those previously used to transfer single and multiple [{MTCPP}Zn_2_(H_2_O)_2_] (M = Cu, VO) Langmuir layers on Si and Mylar substrates.^29,30^ Nanodomains form at the air–water interface of a Langmuir trough upon spreading [VO(H_4_TCPP)] on an aqueous ZnCl_2_ subphase, compression to a surface pressure of 5 mN/m and horizontal-dipping of the chip with the 10 LERs. Four successive transfers were done. On basis of the bulk material structure each 2D MOF plane would provide a surface spin density of ca. 3.46 × 10^13^/cm^2^, if true monolayers are transferred.

**Figure 2:**
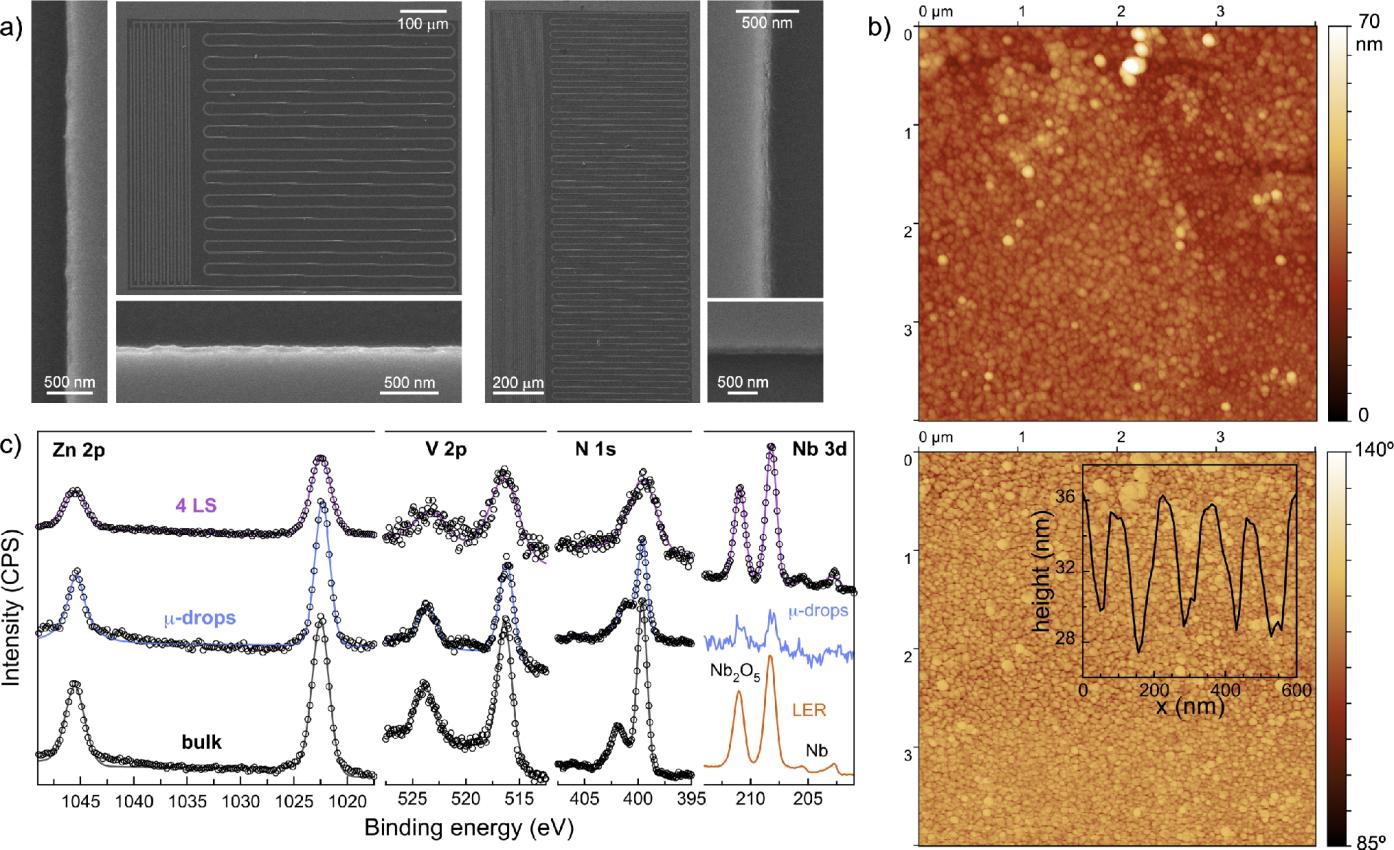
(a) SEM images of LER5 (left) and LER8 (right) covered with a 4-layer LS deposit of [{VOTCPP}Zn_2_(H_2_O)_2_], together with higher magnification images of vertical and horizontal Nb lines showing the continuous LS film. (b) AFM topography (top) and phase (bottom) images together with a typical height profile (inset) of the 4-layer LS deposit of [{VOTCPP}Zn_2_(H_2_O)_2_] on a portion of a Nb line from LER8. (c) High resolution XPS spectra measured on the 4-layer LS deposit on LER8, compared to those of the bulk material and of a thicker deposit grown from μ-droplets,^30^ as indicated. Solid lines represent the best fit envelope spectra.

Scanning electron microscopy (SEM) images (Figures 2a, S8) show that the [{VOTCPP}Zn_2_(H_2_O)_2_] film covers homogeneously the whole chip, despite the complex pattern of ca. 0.5 μm thick superconducting Nb lines. Higher magnification images confirm that the layer is continuous, with hardly any breaks. Importantly, this implies that the film also covers the Nb line edges, which concentrate the photon magnetic field.^15−17,25^ AFM images also show a full coverage of the chip’s surface by 60–100 nm wide rounded nanodomains (Figure 2b), very similar to those previously reported on Si or mylar.^30^ The fact that the film is made of relatively small domains, likely confers to it sufficient flexibility to fully cover the chip without breaking, despite the latter large and sharp variations in height (Figures S9 and S10).

XPS was used to study the chemical nature of the film. The survey spectrum is found to be virtually identical to those obtained for the bulk material and for a thicker deposit grown by 34 LS transfers on Mylar. The only difference is the presence of Nb and Si bands that correspond to the chip surface (Figure S11). Figure 2c compares high resolution Zn 2p, V 2p and N 1s spectra measured on the present film on LERs with those of a thicker deposit grown from μ-droplets, and of the bulk.^30^ The analysis of these spectra leads to very similar bonding energies for the three samples (Table S1). The only significant difference is that the absorption lines of the deposits are broader than in bulk, more so for the thinner LS deposit. Likely, this results from the reduced thickness and the ensuing stronger interaction with the substrate. Importantly, while both orientations of the V=O group are present in the 2D MOF planes, only one set of V 2p signals is observed. This means that V=O groups from the bottom layer having the “oxygen-down” and “oxygen-up” orientations do not have a significantly different interaction with the superconducting surface, in contrast with the strong orientation dependence that was observed for isolated vanadyl phthalocyanine molecules grafted onto a perfectly flat metallic Pb(111) surface.^40^

Overall, there is no indication that the electronic properties of the vanadyl moieties are strongly affected by the proximity of the chip surface, but the presence of two bands at 399.6 and 400–402 eV in the N 1s spectra deserves some attention (Table S1, Figure S12). While this could be interpreted as a mixture of metalated and free-base porphyrins, the estimated proportion of the latter would be 20–30%,^41^ which is in complete disagreement with the UV–vis spectrum of LS deposits on Mylar (Figure S13). Also, the minor band is too strong to be due to the shakeup component of the main one. The observation of two bands in the N 1s spectrum of a metalloporphyrin can also be due to some kind of interaction with the environment that is able to break the local symmetry.^42^ Weak supramolecular interactions among the 2D planes involving the vanadyl moieties and/or the porphyrin aromatic core, observed in the structure of the bulk material, could give rise to this effect.

Then, the reduced separation among the two bands in the two types of deposit could reasonably be due to the reduced number, and lateral sizes, of the 2D planes that form each nanodomain. Figure 2c also compares the XPS Nb 3d spectrum measured on a bare LER with those of the 4-layer LS and μ-droplets deposits. The intensity ratio between the peaks corresponding to metallic Nb and to Nb_2_O_5_ confirms that the outer surface is made of native Nb oxide.^43^ Besides, the overall similar Nb peaks become hardly visible in μ-droplets deposits, thus confirming that these films are then much thicker.

**Figure 3:**
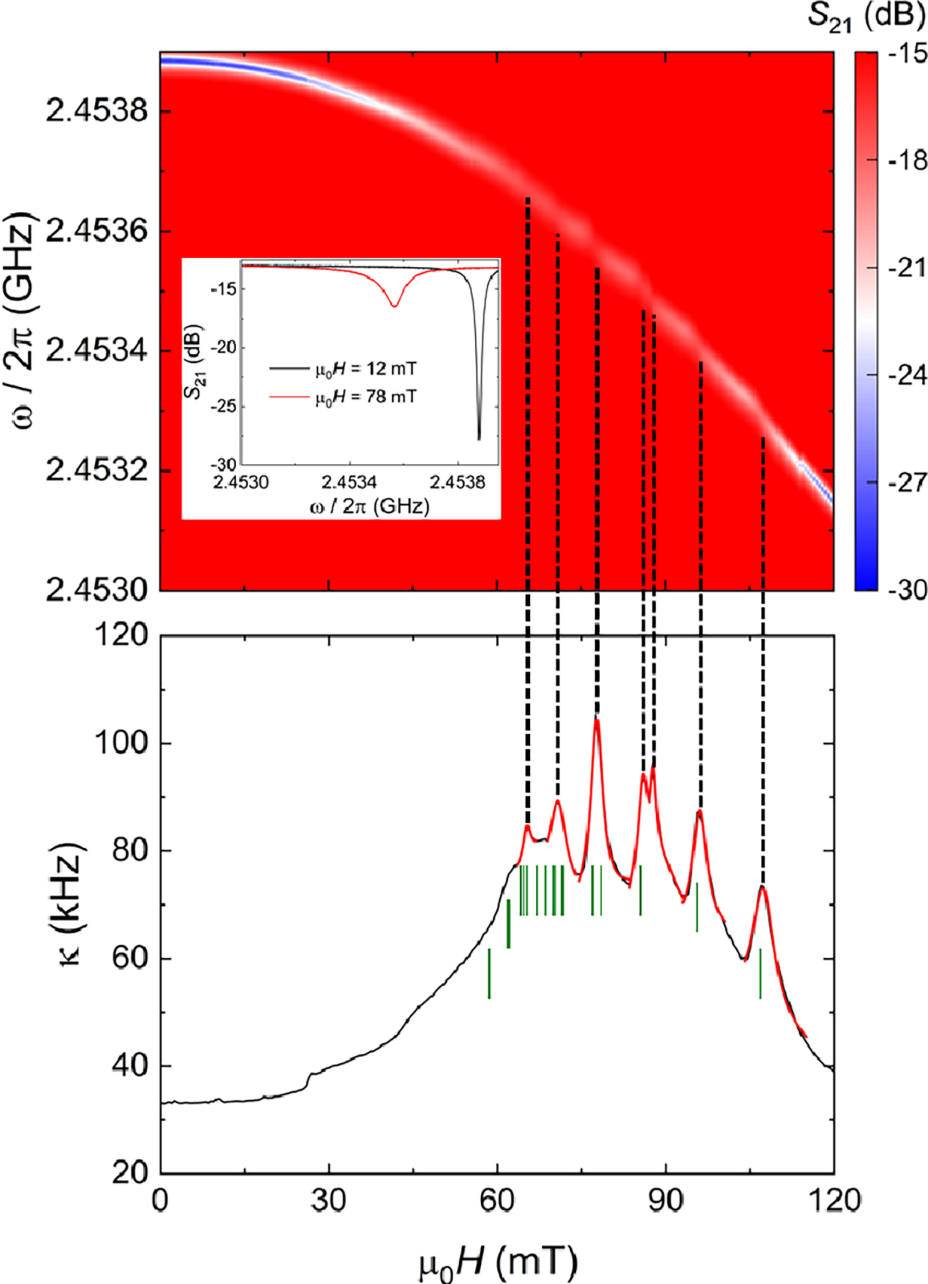
Color plot of the microwave transmission as a function of magnetic field measured at 10 mK near the bare resonance (2.455 GHz) of LER5 covered with ca. 1.10 × 10^12^ vanadyl spins (top) and the corresponding field dependence of the resonator line width (bottom). The inset shows the reduced resonator visibility at a field where resonant coupling with a vanadyl transition takes place. Vertical dashed lines highlight the dips in visibility associated with each clearly defined line width maxima. Green vertical ticks mark the fields at which a resonant spin transition is predicted to occur for the vanadyl spins when the magnetic field is applied perpendicular to its *z* molecular axis. Red lines are least-squares fits of each maxima based on the weak coupling eq 1.

The thickness of the [{VOTCPP}Zn_2_(H_2_O)_2_] nanodomains can be estimated from the analysis of AFM images, as those shown in Figure 2b. It is found to be homogeneous over large areas and ca. 5–6 nm, slightly larger than previously observed on Mylar.^30^ This indicates that each nanodomain is made of few, e.g., 3–4, MOF 2D planes. Considering their rounded shape and size, the nanodomains can be roughly taken as ellipsoids with high aspect ratios. Although these can in principle reach a maximal packing density of 0.77,^44^ a reasonable higher range packing density for shapes similar to our nanodomains with some size distribution is rather 0.70.^45^ Thus, and assuming a similar uniform coverage is maintained over the four successive LS transfers, as previously observed on Mylar,^30^ the film is equivalent to at most 10 MOF 2D planes, on average. This translates into each LER being covered by at most 3.46 × 10^14^ spins/cm^2^, i.e., respectively 1.10 × 10^12^ and 3.09 × 10^12^ spins on the sensitive inductor part of LER5 and LER8 (considering their respective areas, see Figure S1).

### Microwave Transmission Experiments

Transmission experiments were performed by sending a highly attenuated microwave signal to the input port of the chip and measuring the output by a vector network analyzer as a function of frequency and magnetic field. The chip was fixed to a coldfinger, in thermal contact with the mixing chamber of a cryo-free dilution refrigerator (8 mK ≤ *T* ≤ 800 mK), at the center of a 1 T superconducting magnet. We focus here more particularly on LERs number 5 and 8, whose frequencies are shown as horizontal lines in Figure 1a. Because all the resonators are coupled to the same common central line, their resonances give rise to transmission dips at their given frequencies, as shown in Figure 1d. The resonant coupling to any vanadyl electronuclear spin transition is expected to increase the effective resonator line width κ and reduce the resonator visibility (i.e., the maximum change in *S*_21_ at resonance, see inset in Figure 3). Figure 3 shows the results obtained for LER5 at 10 mK where, indeed, this is clearly observed in the magnetic field range at which several electronuclear spin resonances of the [VOTCPP] node match *ω*_*r*_. Quite remarkably, the field positions at which maxima in *k* are observed are in excellent agreement with those calculated for the vanadyl group in bulk [{VOTCPP}Zn_2_(H_2_O)_2_] for κ perpendicular to the vanadyl axis (green vertical segments in Figure 3 bottom). This shows that the orientation of the porphyrin plane in the LS film is quite homogeneous and parallel to the LER surface, and confirms the advantage of using nanodomains of 2D MOF to control the orientation of anisotropic spins.^30^

These experiments can be analyzed to estimate the collective spin-photon coupling *G*_N_ for each resonant transition. For this, we fit the field dependence of *κ* with eq 1, valid for cases where *G*_N_ is much smaller than the spin line width Γ = (*T*_2_*)^−1^.
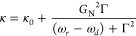
1where *ω*_*r*_ and *ω*_*d*_ are, respectively, the LER frequency and the frequency of the external microwave driving sent to the readout line. The fits, shown as red lines in the bottom panel of Figure 3, provide an estimate of *G*_N_ and Γ. We find *G*_N_/2π values of, respectively, 1.3, 1.2, and 1.6 MHz for the maxima at 80, 95, and 110 mT, while for the other transitions *G*_N_/2π remains in the range 0.5–1.1 MHz. The spin line width Γ is found to be in the 20–70 MHz range. These values compare reasonably well with the inhomogeneous broadening expected for such concentrated system, in which dipole–dipole interactions are not very weak. The shortest vanadyl–vanadyl separation within the 2D planes is 1.7 nm,^30^ which corresponds to a dipolar energy scale μ_B_^2^/*d*^3^ ∼ 2.8 MHz. Vanadyl moieties from adjacent molecular planes can be as close as 0.7 nm, depending on the packing,^30^ which corresponds to a dipolar energy scale ∼39.5 MHz. In addition to these relatively narrow maxima, *κ* shows also a broader background signal, which might point out to some spins having a higher inhomogeneous broadening, which we tentatively associate with a *g*-strain (see below).

Similar measurements have also been performed at increasing temperatures up to 600 mK (Figure S14), to explore the effect of variation in the populations of the different spin levels. The collective spin-photon couplings tend to decrease with increasing temperature for all spin transitions (Figure S15), as expected due to the decreasing population of the ground electronic spin state. Meanwhile, the spin line widths Γ remain approximately constant over the full temperature range (Figure S16). Yet, there seem to be deviations from the thermal equilibrium behavior below approximately 0.1 K. This might indicate that the time needed to restore the equilibrium level populations then approaches, and eventually overcomes, the time it takes to perform each transmission experiment, i.e., several seconds or even minutes. This effect might account for the lower-than-expected intensities observed for the two lowest field resonances. It also agrees with the long spin–lattice relaxation times *T*_1_ derived from pulsed-EPR experiments on the bulk [{VOTCPP}Zn_2_(H_2_O)_2_] material and by μ-Hall measurements on the [VOTCPPEt] precursor, respectively 11 ms at 6 K,^30^ and 9 s at 0.4 K.^19^ Clearly, although a sufficiently long *T*_1_ is beneficial, as it sets the maximum achievable coherence times for isolated spins, such very long relaxation times, typical of vanadyl moieties,^46−49^ represent a drawback for an efficient qubit initialization.

**Figure 4:**
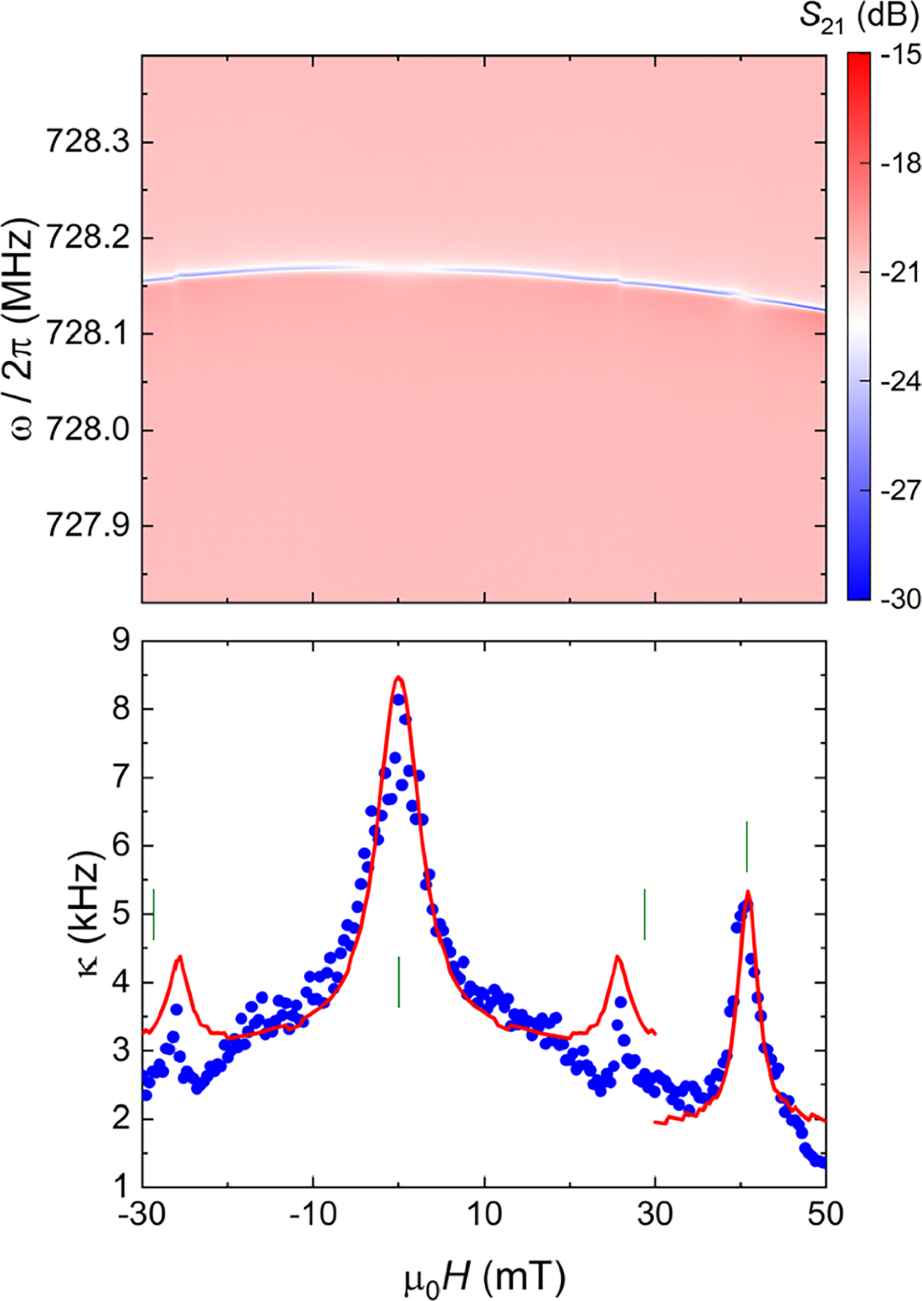
Color plot of the microwave transmission as a function of magnetic field measured at 10 mK near the bare resonance (0.728 GHz) of LER8 covered with ca. 3.09 × 10^12^ vanadyl spins (top) and the corresponding field dependence of the resonator line width (bottom). Green vertical ticks correspond to the spin resonance fields calculated for the vanadyl spins for a magnetic field applied perpendicular to its molecular *z* axis. Red lines are fits of each maximum based on eq 1.

The results of similar experiments for LER8 at 10 mK are shown in Figure 4. The frequency of this resonator is tuned to transitions resonating at lower magnetic fields, taking place between states that exhibit a higher degree of electronuclear spin entanglement. Again, maxima in k and minima in transmission visibility are clearly observed at the magnetic resonant fields expected for these transitions, namely the 7 → 10 clock transition at zero-field and transitions 8 → 11 and 8 → 9 (see Figure 1a), at respectively 26 and 41 mT. The background signal is much smaller, in relative amplitude, for these transitions than for those measured at higher magnetic fields (bottom of Figure 3). This supports the idea that the main source of broadening in these LS films, besides spin–spin interactions, arises from g-strain.

The collective spin-photon couplings *G*_N_/2π determined from fits of *κ* based on eq 1 are respectively 0.10, 0.08, and 0.32 MHz, while *T*_2_* = Γ^–1^ values are 7, 5, and 5 ns. The same transitions are detected with LER7, which has a slightly higher frequency, at 27, 34, and 50 mT with larger *G*_N_/2π ca. 0.55, 0.16, and 0.67 MHz, respectively (Figure S17).

The strong collective coupling regime, defined by *G*_N_ being larger than the spin decoherence rate and κ, is not achieved here, since *G*_N_ is significantly smaller than the spin line width Γ in all cases. This is nevertheless a direct consequence of the significantly reduced number of spins, by various orders of magnitude with respect to previous studies where the strong collective coupling regime was reached.^24^ Indeed, , with N being the total number of spins coupled to the LER and *G*_1_ being the average single spin coupling strength. Despite this, the high cooperativity regime, in which every photon is coherently transferred to the spin ensemble thereby allowing readout, is almost reached. Indeed, the cooperativity  is found to be up to ca. 0.6, thus close to 1. This is very encouraging, considering that Γ could be easily reduced by working with diluted nanodomains, as done previously on the bulk 2D MOF.^30^

Estimating the single spin-photon coupling  first requires a correct estimation of N. Simulations of the photon magnetic field for LER5 show that the coupling of vanadyl spins in the valleys between the inductor lines is negligible (Figure S18), which means only those vanadyl spins on top of the inductor lines will actually couple to the photon(s). Considering the topology of the inductor, with 4 μm wide lines and 21 μm separation between lines, N is actually only ca. 4/25 the total number of spins on the inductor area determined above. For LER5, this then gives single spin-photon couplings in the 1.3–3.9 Hz range. This is up to over 7 times higher than what we previously measured for a thicker deposit grown from μ-droplets on a 1.34 GHz coplanar resonator.^30^

## Conclusions and Outlook

We have shown the tuning of superconducting lumped-element resonators to different groups of electronuclear transitions of a vanadyl porphyrin spin qudit. The reliable fabrication process and the obtained resonators characteristics, in particular their *Q*_*i*_ values up to 2 × 10^6^, i.e., photon life times longer than 40 μs, validate their use to couple molecular spins to resonant photons. To dispose a controlled number of spin qubits at specific locations in superconducting resonators, the *in situ* formation of nanodomains of the 2D [{VOTCPP}Zn_2_(H_2_O)_2_] MOF, that contains the same vanadyl node, has been explored using Dip-Pen nanolithography. Although a successful process, its localization can only be efficiently controlled when it is done within the confined area of μ-wells made on Si substrates. In this respect the nanolithography of preformed NPs or nanocrystals appears better suited.^28^

Monolayers of nanodomains of the same MOF, formed at the air–water interface, were then subsequently transferred to a chip with 10 frequency-tuned LERs through the Langmuir–Schaefer technique. The sequential nature of the method allows to adjust the number of spins per surface area by controlling the number of transfers. Here four successive transfers resulted in an ultrathin film of ca. 20–24 nm thickness and a density of ca. 3.46 × 10^14^ spins/cm^2^ that fully covers the whole LERs, including their Nb edges that concentrate the photon magnetic field. Transmission experiments then show the efficient coupling of photons to the targeted electronuclear transitions. The excellent agreement of the magnetic fields at which these transitions are detected with theoretical predictions confirms that the orientation of the vanadyl porphyrin node in the film is homogeneous and parallel to the chip surface, thanks to the subjacent regular framework. These experiments allowed to derive values of collective spin-photon couplings *G*_N_ in the range 0.5–1.6 MHz. Although not reaching the (collective) strong coupling regime, the estimated single spin-photon couplings, in the range 1.2–3.9 Hz, are significantly improved with respect to those previously measured with a coplanar resonator,^30^ confirming the enhanced microwave magnetic field provided by LERs. Also, the high cooperativity regime, in which every photon is coherently transferred to the spin ensemble, is almost reached, despite the reduced number of spins and the reduced *T*_2_* values resulting from the concentrated nature of the nanodomains.

The results suggest that a proper interface between quantum circuits and a regular array of spin qudits can be achieved. Within this approach, the ability of addressing fewer, or even individual nodes from the array relies on the proper definition of “hot” spin-photon coupling spots, which can likely be done via the combination of a suitable circuit design and nanopatterning. A number of improvements should allow reaching stronger spin-photon couplings and cooperativity. On one hand, improved resonators such as Au/Nb LERs^50^ and/or the use of constrictions can provide much enhanced photon magnetic field and thus *G*_N_. On the other hand, both dilution with diamagnetic nodes and/or using qubits nodes with better coherence would directly enhance the efficiency of the spin-photon coupling, since it is ultimately controlled by the spin decoherence rate. In this respect, another method allowing full coverage with molecular layers of qubits and homogeneous orientation that uses a modular approach combining self-organization and click chemistry^51^ could open up the library of qubits that can be integrated.

## Supporting Information Available

All experimental details and methods; AFM, SEM and Raman spectroscopy characterization of deposits made by sequential DPN; additional SEM, AFM and XPS characterization of 4-layer Langmuir–Schaefer film on LERs; UV–vis spectra; additional microwave transmission data; simulation of photon magnetic field on LER5 (PDF)
