# Supplementary material for: Localized Nanoscale Formation of Vanadyl Porphyrin 2D MOF Nanosheets and Their Optimal Coupling to Lumped Element Superconducting Resonators
Source: J Phys Chem C Nanomater Interfaces. 2024 Dec 25;129(1):973–82. doi: 10.1021/acs.jpcc.4c07265 (PMC11726679; doi:10.1021/acs.jpcc.4c07265)
Supplement: Supplementary file 1 — jp4c07265_si_001.pdf [file jp4c07265_si_001.pdf]

Supporting information for the manuscript

# Localized Nanoscale Formation of Vanadyl Porphyrin 2D MOF Nanosheets and their Optimal Coupling to Lumped Element Superconducting Resonators

Ignacio Gimeno,[a] Fernando Luis,[a] Carlos Marcuello,[a,b,‡] Maria Carmen Pallarés,[a,b] Anabel Lostao,[a,b,c] Marina Calero de Ory,[d] Alicia Gomez,[d] Daniel Granados,[e] Inés Tejedor,[a] Eva Natividad,[f] Ainhoa Urtizberea,[f] Olivier Roubeau[a]

*a Instituto de Nanociencia y Materiales de Aragón (INMA), CSIC – Universidad de Zaragoza, Plaza San Francisco s/n, 50009, Zaragoza, Spain. E-mail: roubeau@unizar.es, fluis@unizar.es*
*b Laboratorio de Microscopias Avanzadas (LMA), Universidad de Zaragoza, Ed. I+D+i. Mariano Esquillor s/n, 50018 Zaragoza, Spain*
*c Fundación ARAID, Av. Ranillas 1-D, 50018 Zaragoza, Spain*
*d Centro de Astrobiología, CSIC – INTA, Torrejón de Ardoz, 28850 Madrid, Spain. E-mail: agomez@cab.inta-csic.es*
*e IMDEA Nanociencia, Cantoblanco, 28049, Madrid, Spain*
*f Instituto de Nanociencia y Materiales de Aragón (INMA), CSIC – Universidad de Zaragoza, Campus Rio Ebro, María de luna 3, 50018 Zaragoza, Spain*
*‡ Current affiliation: Biofisika Institute (CSIC, UPV/EHU), 48940 Leioa, Spain*

## Table of contents

| | |
|---|---|
| *Experimental details* | p. S2 |
| Figure S1. Schematic and optical views of the chip hosting the 10 LERs | p. S6 |
| Figure S2. In-situ DPN deposition and negative control on bare Si | p. S7 |
| Figure S3. SEM and AFM of a representative $\mu$-well on Si | p. S8 |
| Figure S4. AFM and Raman spectroscopy of [{VOTCPP}$Zn_2(H_2O)_2$] domains formed through sequential DPN lithography in $\mu$-wells | p. S9 |
| Figure S5. DPN in-situ deposition process $\mu$-well on Si | p. S10 |
| Figure S6. In-situ DPN deposition in $\mu$-well on Si | p. S11 |
| Figure S7. Negative control for DPN deposition in $\mu$-well on Si | p. S12 |
| Figure S8. SEM of LER5, LER7 and LER8 covered with the LS deposit | p. S13 |
| Figure S9. AFM observations of the 4 LS deposit on the Nb line of LER8 | p. S14 |
| Figure S10. AFM observations of the 4 LS deposit on LER8 | p. S15 |
| Figure S11. XPS survey spectrum of the LS film on LER5 | p. S16 |
| Table S1. Parameters for the simulation of high-resolution XPS spectra. | p. S17 |
| Figure S12. High resolution XPS N 1s spectra and their simulation | p. S18 |
| Figure S13. UV-Vis spectra | p. S19 |
| Figure S14. Microwave transmission of LER5-LS film and corresponding field dependence of the linewidth at increasing temperatures. | p. S20 |
| Figure S15. Temperature dependence of $G_N/2\pi$ for LER5-LS film | p. S21 |
| Figure S16. Temperature dependence of the line width of the maxima in the field dependence of $\kappa$ for LER5-LS film | p. S22 |
| Figure S17. Microwave transmission of LER8-LS film and corresponding field dependence of the linewidth at 10 mK | p. S23 |
| Figure S18. Simulation of photon magnetic field on LER5 | p. S24 |
| References | p. S25 |

## Experimental details

### Physical characterization

*Raman spectra* were obtained with a WITec Alpha 300 (Oxford Instruments) equipped with a confocal microscope (INMA, CSIC and Universidad de Zaragoza). The laser used was 532 nm. Measurements were made with 0.5 mW power to avoid burning the metal-organic samples. Besides full Raman spectra at various positions of each samples, a mapping of the deposits made in m-wells (see below) was done at 1590 $cm^{-1}$ with a resolution of 32 pixels/line.

*Scanning Electron Microscopy (SEM)* observations were made by using an INSPECT-F50 instrument (LMA, Universidad de Zaragoza), both operating at an accelerating voltage of 10 keV. Samples were previously sputter-coated with 14 nm of Pd.

*X-ray Photoelectron Spectroscopy (XPS)* measurements were performed in a Kratos AXIS SUPRA spectrometer at the Laboratorio de Microscopías Avanzadas (LMA, Universidad de Zaragoza), using a monochromatized Al K$\alpha$ source (1486.6 eV). Wide scans were acquired at an analyser pass energy of 160 eV, whereas high-resolution narrow scans were performed at constant pass energy of 20 eV. The spectra were obtained at room temperature. The binding energy (BE) scale was internally referenced to the C 1s peak (BE for CC=284.9 eV).

*Atomic Force Microscopy (AFM)* characterization of all DPN deposits was performed with a Multimode V SPM setup equipped with Nanoscope V Controller (Bruker; LMA, Universidad de Zaragoza) using 320 kHz nominal resonant frequency rectangular silicon TESPA-V2 cantilevers (Bruker, Santa Barbara, CA, USA). Characterization of the 4 LS deposit on the chip with Lumped Element Resonators was performed on a Nano-Observer (CSI Instruments; INMA, CSIC and Universidad de Zaragoza). FORTA cantilevers (AppNano) with a nominal resonance frequency of 61 kHz were used. All observations were done in air under ambient conditions working in tapping (Bruker) oscillating (CSI) mode. Raw images were analysed with the Gwyddion software tool.[1]

*Transmission experiments* were performed by mounting the chip with Lumped Element Resonators covered with by 4 LS ultra-thin film in a BlueFors LD450 dilution refrigerator equipped with a 1 T superconducting magnet (INMA, CSIC and Universidad de Zaragoza), and connecting it through coaxial cables to a Vector Network Analyzer with a measurement frequency bandwidth ranging from 100 MHz to 14 GHz. Device characterization is then done by measuring the transmitted signal $S_{21}$.

### Reagents

Commercial 5,10,15,20-tetrakis(4-carboxyphenyl)porphine ($H_2$TCPP, >97%) was purchased from TCI. $VO(SO_4)\cdot H_2O$ (>99.0%), $ZnCl_2$ (>98%), $Zn(NO_3)_2\cdot 6H_2O$ (98%), glacial acetic acid and reagents/HPCL grade solvents N,N'-dimethylformamide (DMF), chloroform, dichloromethane, ethanol and methanol were purchased from Aldrich and used without further purification.

### Synthesis of [VOTCPPEt], [VOTCPP] and [{VOTCPP}$Zn_2(H_2O)_2$]

This was done as previously described.[2] Briefly, [VOTCPPEt] was first obtained by reacting 5,10,15,20-tetracarboxyphenylporphyrin ($H_2$TCPP) with $VOSO_4\cdot xH_2O$ in

ethanol under solvothermal conditions at 160ºC for 24h. Then [VOTCPP] was obtained by reacting [VOTCPPEt] in dichloromethane/methanol with sodium hydroxide, and then reacting the obtained solid with glacial acetic acid. Eventually, [{VOTCPP}$Zn_2(H_2O)_2$] is obtained by reacting [VOTCPP] with $Zn(NO_3)_2\cdot 6H_2O$ in DMF under solvothermal conditions at 124ºC for 50h.

### Substrates for DPN deposition

Silicon wafers of 525 µm thickness and oriented along <100> were used without specific treatment, *i.e.* the native $SiO_2$ layer is present in all cases, and the substrates are thus rather Si/$SiO_2$.

### Microfabrication of marks and microwells on Si substrates

Marked Si substrates were first treated with dual beam Helios Nanolab 650 (FEI, USA; LMA, Universidad de Zaragoza) to create a pattern of letters using focused ion beam (FIB), which was later coated by gold evaporation. The setup is coupled with a SEM to conduct a first quality control of the produced wafer. The same dual beam system was also used to create holes on the previously marked substrates. The beam acceleration was fixed at 30 kV and 9.3 nA with several exposure times of 5 min to create circular wells of 25 to 100 µm diameter and 50 to 200 nm depth separated by 200 to 300 µm. The electronic lithography was developed using the Raith software (GmbH, Germany).

### *In-situ* formation of [{VOTCPP}$Zn_2(H_2O)_2$] nanodomains using DPN

A solution of 0.12 mmol/L (0.1 mg/mL) of [VO(TCPP)] was prepared in $CHCl_3$:$CH_3OH$ (3:1, v/v), while 1 and 100 mmol/L aqueous solutions of respectively $CuCl_2$ and $ZnCl_2$ were prepared using Milli-Q water (ρ = 18.2 MΩ·cm). The deposition was performed with a DPN5000 setup (NanoInk, Inc.; INMA, CSIC and Universidad de Zaragoza) using silicon nitride ORC8 rectangular cantilevers for contact exhibiting 0.1 N/m stiffness (Bruker Probes). DPN experiments were carried out at room temperature under 60% relative humidity. The transfer process is divided in two different steps, for which two cantilevers are used to be coated with the two solutions. The procedure to functionalize each pen consists on the immersion of the tip several times on a 0.1 µL drop poured with a Gilson micropipette on a different place of the same substrate. Firstly, a droplet of $ZnCl_2$ or $CuCl_2$ aqueous solution is deposited by the pre-coated AFM tip on either the bare Si or within a µ-well. Then, the other probe wetted with the [VO(TCPP)] solution is placed on the external surface of the salt drop previously deposited. The contact time is 2 minutes to ensure the proper transfer of porphyrins on the well surface and their subsequent coordination with the Cu(II) or Zn(II) ions present in the aqueous “subphase”. After full evaporation, the silicon wafer is gently rinsed with methanol and water in order to remove unreacted porphyrins and excess salt. Finally, the substrate is dried with a mild nitrogen flux.

This protocol mimics *in-situ* the conditions under which MOF nanodomains are formed at the air-water interface of a Langmuir trough, by depositing µ-droplets of a MeOH:$CHCl_3$ solution of [VOTCPP] onto an aqueous $ZnCl_2$ droplet previously deposited over the Nb substrate. Initial attempts were done on a Si wafer with

patterned gold letters in order to localize the sequential lithography deposits (see Fig. S3). The process was done using either $ZnCl_2$ or $CuCl_2$ since the formation of the [{MTCPP}$Cu_2(H_2O)_2$] MOF nanodomains at the air-water interface is more efficient and requires much lower concentration.[2,3] Also, negative controls were done by spreading only the [VOTCPP] ink. After evaporation, the substrate was washed with ultra-pure water and MeOH to ensure removal of unreacted metal salt and free [VOTCPP], and subsequently dried with $N_2$.
AFM images show that some 30-100 nm wide and 2-10 nm high rounded domains appear where the sequential lithography was performed, as opposed to the control areas that only show bare surfaces (Fig. S2). The topography of the nanodomains formed from the air-water interface through the conventional LS technique and of those formed here in-situ are very similar. This points at the likely formation of [{VOTCPP}$M_2(H_2O)_2$] 2D nanodomains.[2] Nevertheless, domains are observed over a much larger area than expected. X-ray Photo-electron Spectroscopy (XPS) shows no sign of the expected elements (Zn or Cu, V, N), which suggests that the density of the material deposited is very low. Although isolated nanodomains seem to be formed *in-situ* through DPN nanolithography, it is clear that their locations cannot be controlled with the required, nanoscopic accuracy. Most likely, this is due to the spreading of the subjacent aqueous nano-droplet over large areas of the flat native $SiO_2$ layer that covers the Si substrate. Since this is intrinsic to the contact angle of the aqueous droplet, similar results can be expected for deposits grown on Nb superconducting circuits, due to the existence of a similar $Nb_2O_5$ native layer (see below).
In order to circumvent this problem, and to allow a proper characterization of the nanodomains, the same procedure was repeated within a physically confined area. For this, $\mu$-wells were fabricated in the Si wafer surface by Focused-Ion Beam (FIB) lithography. These were typically *ca.* 25 or 100 $\mu$m wide and 100 nm deep, and were separated from each other by *ca.* 300 $\mu$m (Fig. S3). Figure S4a-b shows characteristic AFM images of one of these micro-wells taken after the in-situ growth of MOF nanodomains. A continuous coverage of its inner surface by rounded domains is observed, these domains being of similar size and shape as those grown by either conventional LS or by using $\mu$-droplets on flat substrates. Raman spectroscopy was used to confirm the nature of the material formed *in-situ* (Fig. S4c). The spectrum is very similar to that of bulk [{VOTCPP}$Zn_2(H_2O)_2$]. In particular, the characteristic bulk absorption bands at 1020, 1350, 1505 and 1590 $cm^{-1}$ are detected, these being tentatively ascribed to respectively the V=O stretch, the $\nu_4$ and $\nu_2$ modes of the porphyrin core,[4] and the carboxylate group (possibly also $\nu_{CC}$ in phenyl). The absence of the 410 $cm^{-1}$ band, most likely corresponding to a symmetric V-$N_4$ stretching vibration,[5] is compatible with the low signal to noise ratio in this region of the spectrum. It should be pointed out that these spectra are also very similar to that of the precursor molecule [VO($H_4$TCPP)]. Therefore, these data alone cannot provide compelling evidence for the formation of the paddle-wheel $Zn(H_2O)(COO)_4$ nodes. Nevertheless, negative controls when the same procedure is replicated in the absence of Zn(II) or Cu(II) ions do not give rise to any material growth. Therefore, it makes sense to conclude that [{VOTCPP}$M_2(H_2O)_2$] MOF nanodomains are grown in the $\mu$-wells. Also, the strongest Raman band measured at *ca.* 1590 $cm^{-1}$ can be mapped over the whole well (Fig. S4d-e), confirming that nanodomains entirely cover its surface. These results convincingly show that nanodomains of the [{VOTCPP}$M_2(H_2O)_2$] (M = Zn, Cu) MOFs can be formed *in-situ* through DPN under suitable conditions, in this case within a confined region. However, achieving a nanoscopic control over the position of such deposits does not seem to be possible, at least on native oxide surfaces.

**Lumped Element Resonators fabrication**

The nanofabrication of the superconducting niobium resonators was conducted at the Center for Nanofabrication NanoFabLab at IMDEA Nanoscience. The process starts with the pre-treatment of a 275 μm thick silicon (Si) substrate in a 1% hydrofluoric acid bath to remove the native silicon oxide layer. A 100 nm thick niobium (Nb) film is then deposited onto the Si substrate using confocal DC AJA magnetron sputtering, with a chamber's base pressure below $2\times10^{-8}$ Torr. The Nb deposition is carried out at 100 W, with an argon (Ar) pressure of 1.5 mTorr and a flow rate of 15 sccm.

Afterward, maskless laser writer lithography is employed to pattern the chip design onto the Nb film. A layer of negative photoresist (AZ2070) is spin-coated onto the surface and exposed to a 405 nm laser at room temperature. Following the development of the resist, reactive ion etching is performed using a gas mixture of argon (10 sccm, 0.1 mTorr) and sulfur hexafluoride ($SF_6$) (20 sccm, 10 mTorr). The process concludes with the removal of the remaining photoresist by boiling in acetone, followed by an isopropanol rinse.

**Transfer of Langmuir−Schaefer film of [{VOTCPP}$Zn_2(H_2O)_2$] nanodomains**

This was done as previously described for Si, Mylar and quartz substrates,[2] using the chip with Lumped Element Resonators as substrate. For this, a KSV-NIMA trough model KN 2003, with dimensions of 580 mm × 145 mm, housed in a clean room inside closed cabinets and the room temperature is maintained at 293±1 K, was used (INMA, CSIC and Universidad de Zaragoza). The trough was carefully cleaned with acetone and chloroform, filled and emptied twice with Milli-Q water (ρ = 18.2 MΩ·cm), and ultimately filled with a 0.1 M $ZnCl_2$ solution made with Milli-Q water and filtered prior to use on a 0.2 μm membrane to remove the small amounts of Zn oxychloride formed upon solubilization. The subphase was then carefully cleaned by closing the barriers down to 40 mm distance and mild surface-touch vacuuming intra-barriers area. After opening the barriers to the maximum area, the system was let to equilibrium for 5 minutes, and a [VOTCPP] $CHCl_3$:$CH_3OH$ solution (3:1 v:v) was carefully spread drop-by-drop onto the subphase using a Hamilton microsyringe held very close to the subphase surface. After 20 min letting evaporate the organic solvents, compression was performed at constant speed of 7.5 $cm^2\cdot min^{-1}$ until reaching a surface pressure of 5 mN/m. Four successive transfers of the [{VOTCPP}$Zn_2(H_2O)_2$] nanodomains film formed at the air-water interface were then carried out by horizontal-dipping at a surface pressure of 5 $mN\cdot m^{-1}$, the substrate being approached to the surface at 0.2 $mm\cdot min^{-1}$ and raised at 10 $mm\cdot min^{-1}$. Between successive transfers, the substrate was cleaned by gently flushing with Milli-Q water, submerging in Milli-Q water for 3 min, and drying under a $N_2$ flush.

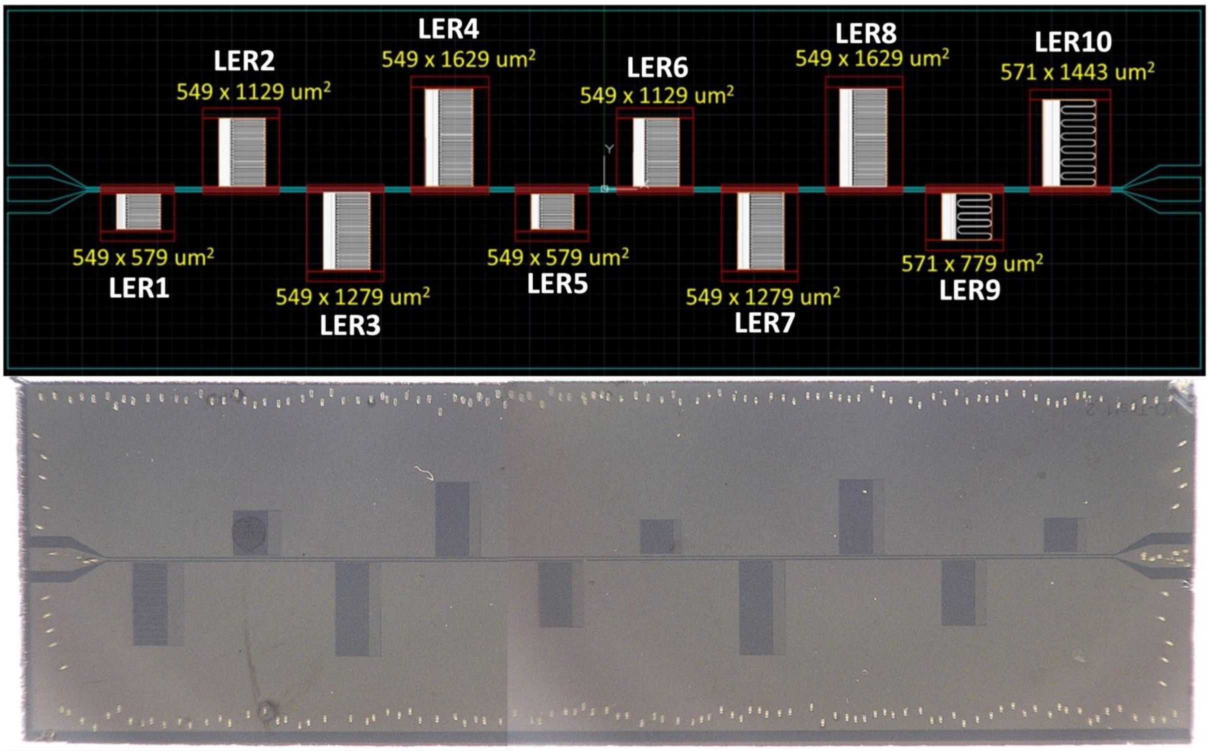


Figure S1. Scheme (top) and optical image (bottom) of the chip hosting the 10 LERs with different design and corresponding frequencies and inductor sizes.

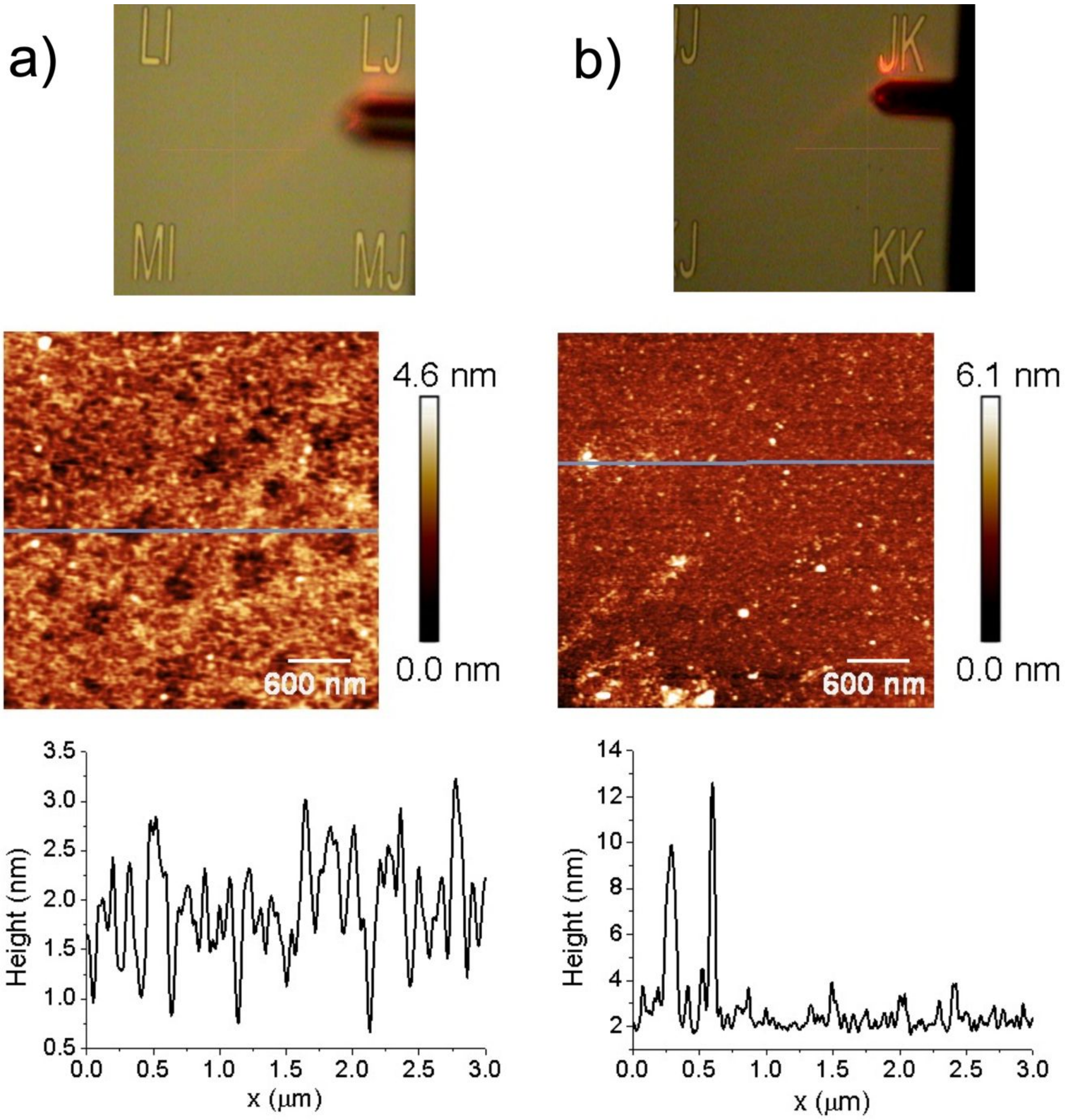


Figure S2. a) Optical image from the DPN system (top) and representative AFM image (middle) and height profile (bottom) of a negative DPN control on bare Si, *i.e.* depositing VOTCPP ink in MeOH/$CHCl_3$ without subjacent $CuCl_2$ aqueous droplet, and after posterior washing and drying. The RMS roughness of the image is 0.65 nm. b) Optical image from the DPN system (top) and representative AFM image (middle) and height profile (bottom) of DPN deposition of VOTCPP ink in MeOH/$CHCl_3$ onto a subjacent $CuCl_2$ aqueous droplet after washing and drying. The RMS roughness of the image is 0.83 nm. The size of AFM images is 3 μm x 3 μm.

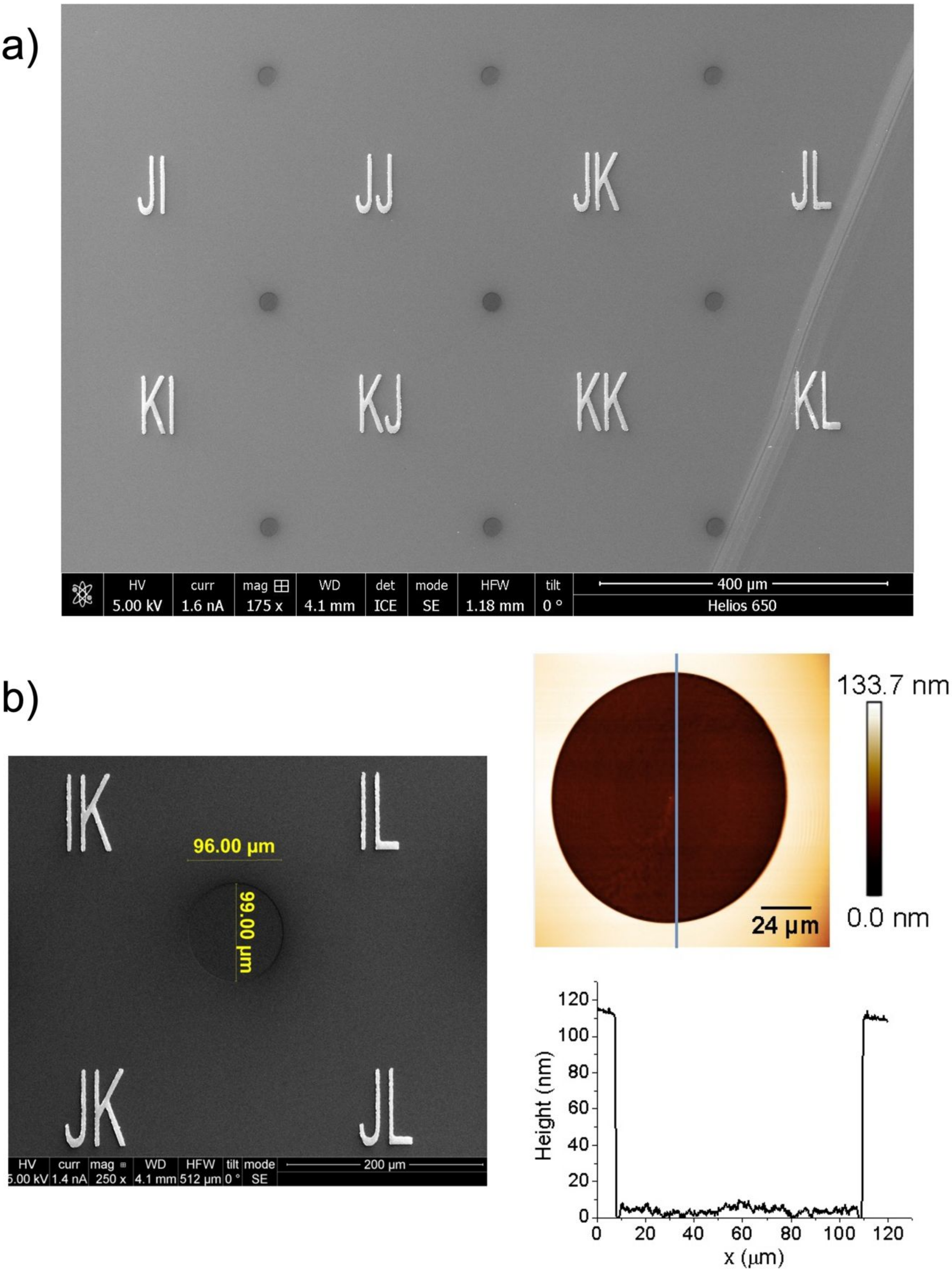


Figure S3. a) SEM image of an array of micro-fabricated wells on Si with 25 μm diameter. b) SEM and AFM images of a micro-fabricated well on Si with 100 μm diameter. The size of the AFM image is 120 μm x 120 μm. The height profile corresponding to the blue line in the image confirms the targeted depth of *ca.* 100 nm.

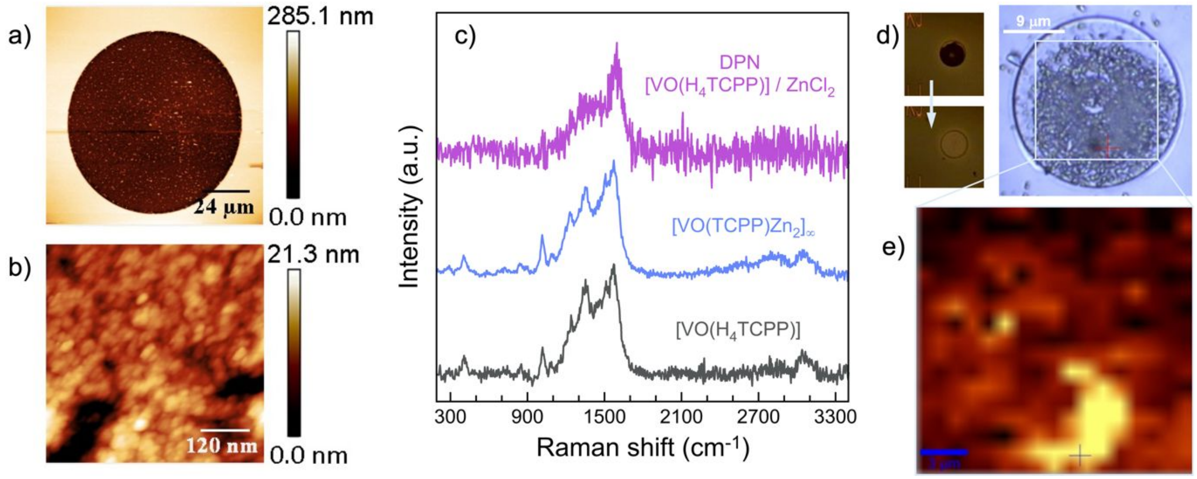


Figure S4. a) and b) AFM images of a μ-well with [{VOTCPP}$Zn_2(H_2O)_2$] grown *in-situ* through sequential DPN lithography. c) Raman spectra of bulk [VO($H_4$TCPP)], bulk [{VOTCPP}$Zn_2(H_2O)_2$] and the [{VOTCPP}$Zn_2(H_2O)_2$] domains formed in-situ through sequential DPN lithography in a μ-well. d) SEM images of a μ-well after DPN deposition of VOTCPP, taken before (top) and after (bottom) washing, and optical image of the same μ-well highlighting the area mapped through Raman spectroscopy. e) Mapping of the Raman 1590 $cm^{-1}$ porphyrin band for an in-well [{VOTCPP}$Zn_2(H_2O)_2$] deposit.

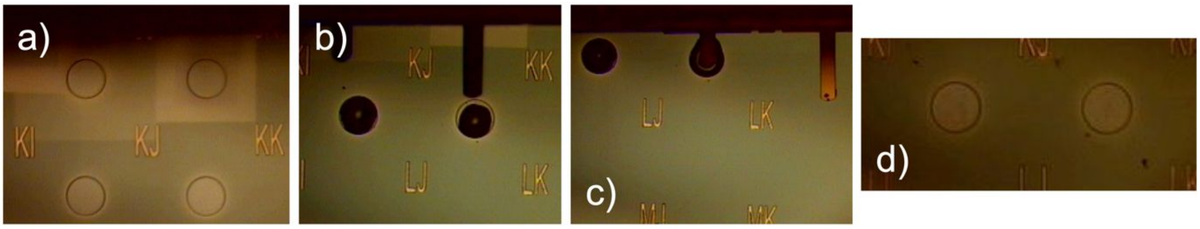


Figure S5. Optical images of the DPN *in-situ* formation of 2D MOF in micro-fabricated wells on Si: a) bare original wells, b) after filling with $ZnCl_2$ aqueous "subphase" (2 deposition steps per well), c) after depositing atop the VOTCPP ink in MeOH/$CHCl_3$ and d) after full evaporation, washing with water and MeOH and drying.

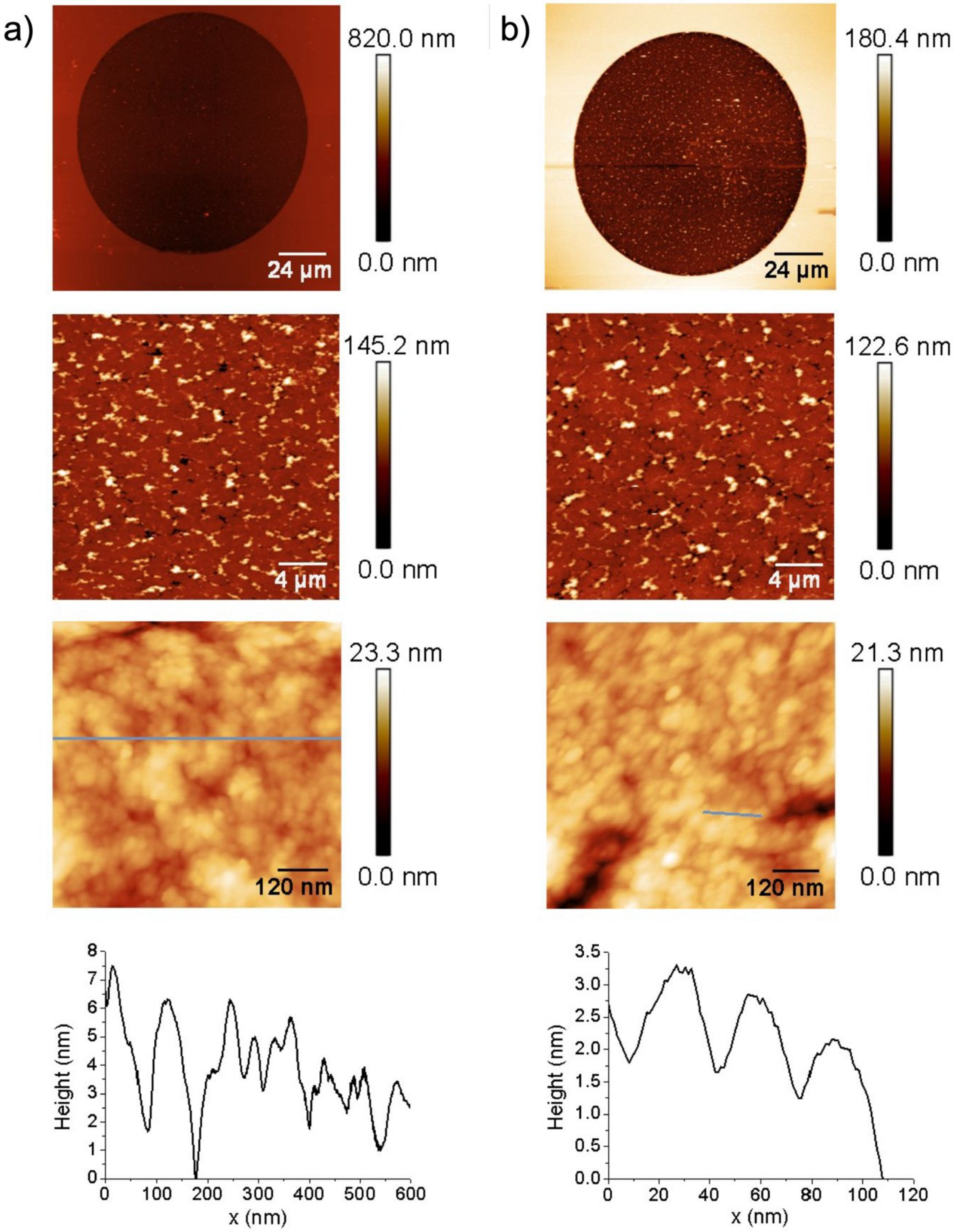


Figure S6. Representative AFM images and height profiles (corresponding to the blue lines in the images) of µ-wells after DPN deposition of VOTCPP ink in MeOH/$CHCl_3$ onto a subjacent $MCl_2$ aqueous droplet (a, M = Cu; b, M = Zn), and after evaporation, washing and drying. Scan sizes are 120 µm x 120 µm, 20 µm x 20 µm and 600 nm x 600 nm, respectively.

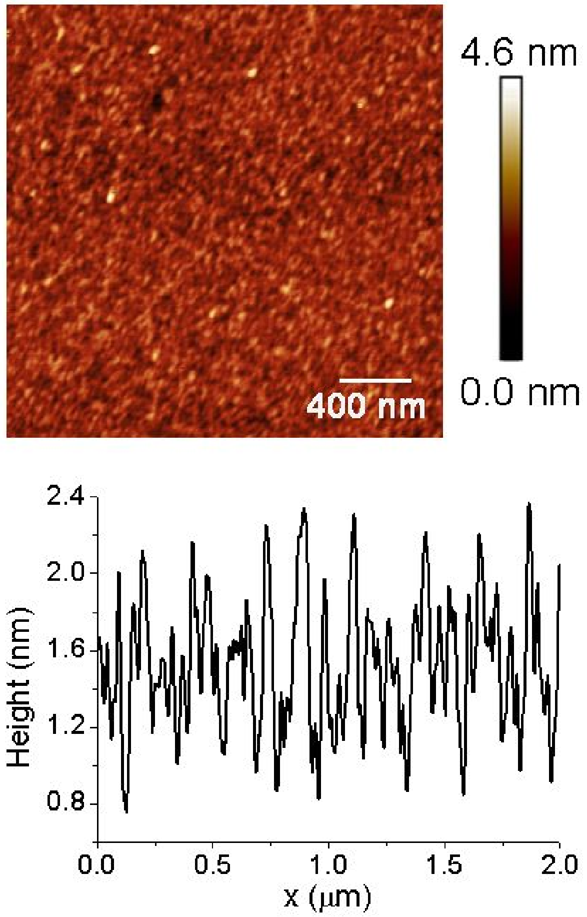


Figure S7. Representative AFM image and height profile of negative DPN control within a μ-well on Si, filling the well with $ZnCl_2$ aqueous droplet without subsequent deposition of VOTCPP ink, after posterior washing and drying. The image size is 2 μm x 2 μm.

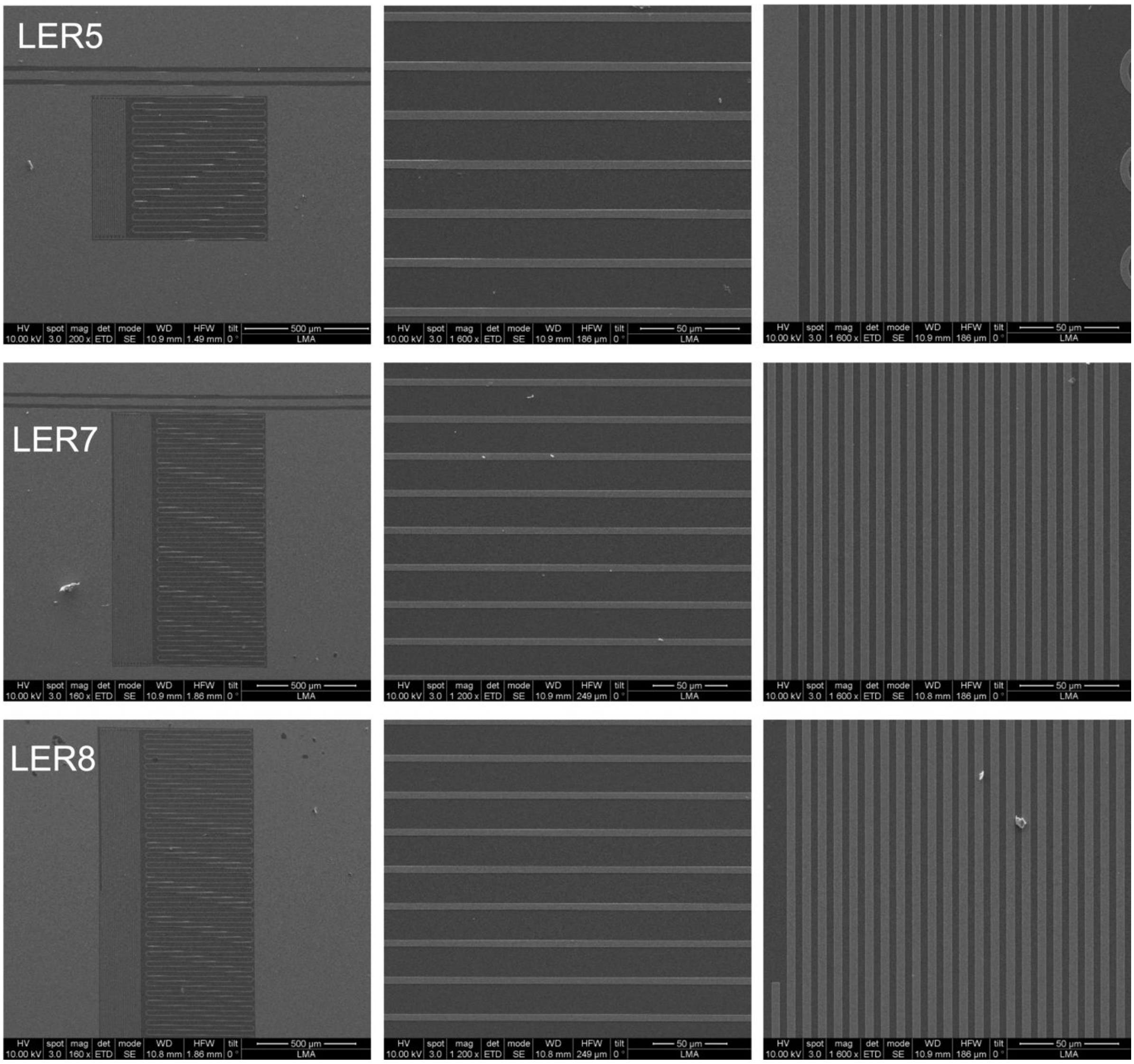


Figure S8. SEM images of LER5 (top), LER7 (middle) and LER8 (bottom) covered with a 4 LS deposit of [{VOTCPP}$Zn_2(H_2O)_2$], showing the full LER (left) and higher magnifications of its L (middle) and C (right) components.

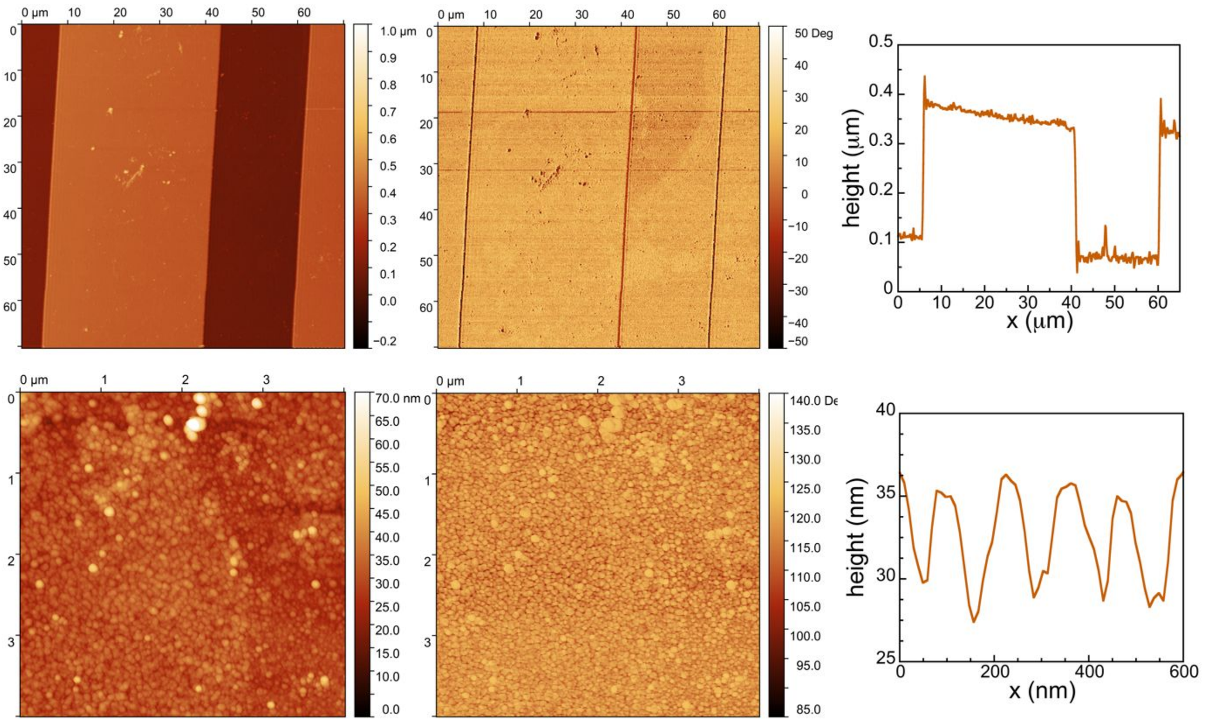


Figure S9. Large (70×70 µm, top) and small (4×4 µm, top) area AFM observations of the 4 LS deposit of [{VOTCPP}$Zn_2(H_2O)_2$] on a portion of a Nb line of LER8: topography (left) and phase (middle) images together with a typical height profile (right).

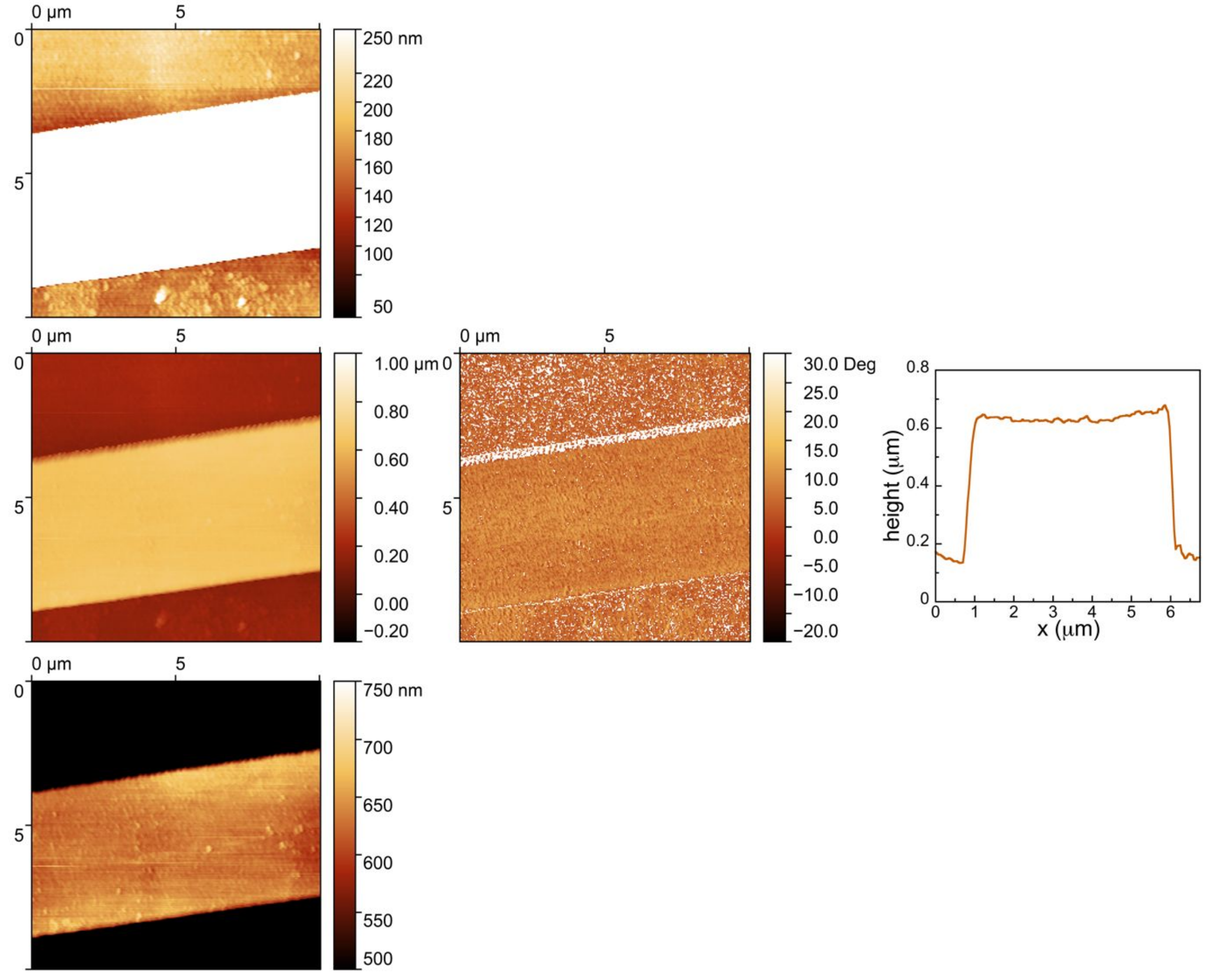


Figure S10. AFM topography image of the 4 LS film of [{VOTCPP}$Zn_2(H_2O)_2$] on a portion of LER8 (left, middle) shown at two additional height scales to highlight how film covers both the bottom Si substrate (left, top) and the Nb line surface (left, bottom). The corresponding phase image (middle, middle) and a characteristic height profile (right, middle) are also shown.

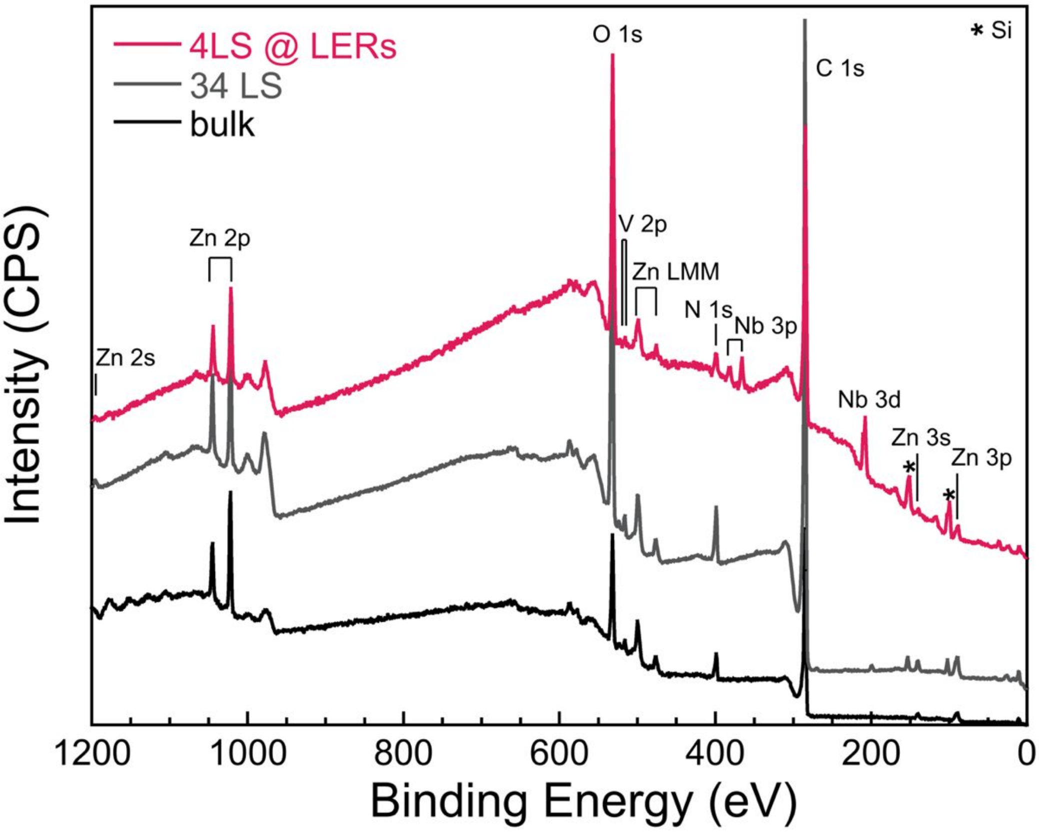


Figure S11. XPS survey spectrum of the 4 LS film of [{VOTCPP}$Zn_2(H_2O)_2$] on LER5 (red line) compared with those previously reported for a 34 LS film on Mylar (grey line) and the bulk material (black line).[2]

Table S1. Parameters used for the simulation of high-resolution XPS spectra of the 4-layer LS deposit on LER8, compared to those previously reported for the bulk material and a deposit grown from μ-droplets on a CPW resonator.[2]

| | **4 LS @ LER8** | | **μ-droplets @ CPW** [2] | | **Bulk** [2] | |
|---|---|---|---|---|---|---|
| | B. E. (eV) | FWHM | B. E. (eV) | FWHM | B. E. (eV) | FWHM |
| Zn 2p | | | | | | |
| 3/2 | 1023.48 | 2.24 | 1022.33 | 1.64 | 1022.40 | 1.85 |
| 1/2 | 1045.51 | 2.40 | 1045.39 | 2.04 | 1045.51 | 2.13 |
| V 2p | | | | | | |
| 3/2 | 516.32 | 3.12 | 516.19 | 1.63 | 516.36 | 1.75 |
| 1/2 | 523.22 | 3.28 | 523.58 | 2.08 | 523.69 | 2.90 |
| N 1s | | | | | | |
| comp. 1 | 399.64 | 1.88 (44.8%) | 399.56 | 0.90 (42.8%) | 398.61 | 1.08 (71.6%) |
| comp. 2 | 401.12 | 2.65 (55.2%) | 400.79 | 2.87 (57.1%) | 400.81 | 1.77 (28.5%) |
| Nb 3d | | | | | | |
| 5/2 ($Nb_2O_5$) | 208.20 | 1.08 (52.4%) | 208.11 | 0.77 | | |
| 3/2 ($Nb_2O_5$) | 210.95 | 1.11 (34.9%) | 210.88 | 0.75 | | |
| 5/2 (Nb) | 202.92 | 1.54 (7.6%) | - | - | | |
| 3/2 (Nb) | 205.72 | 1.47 (5.1%) | - | - | | |

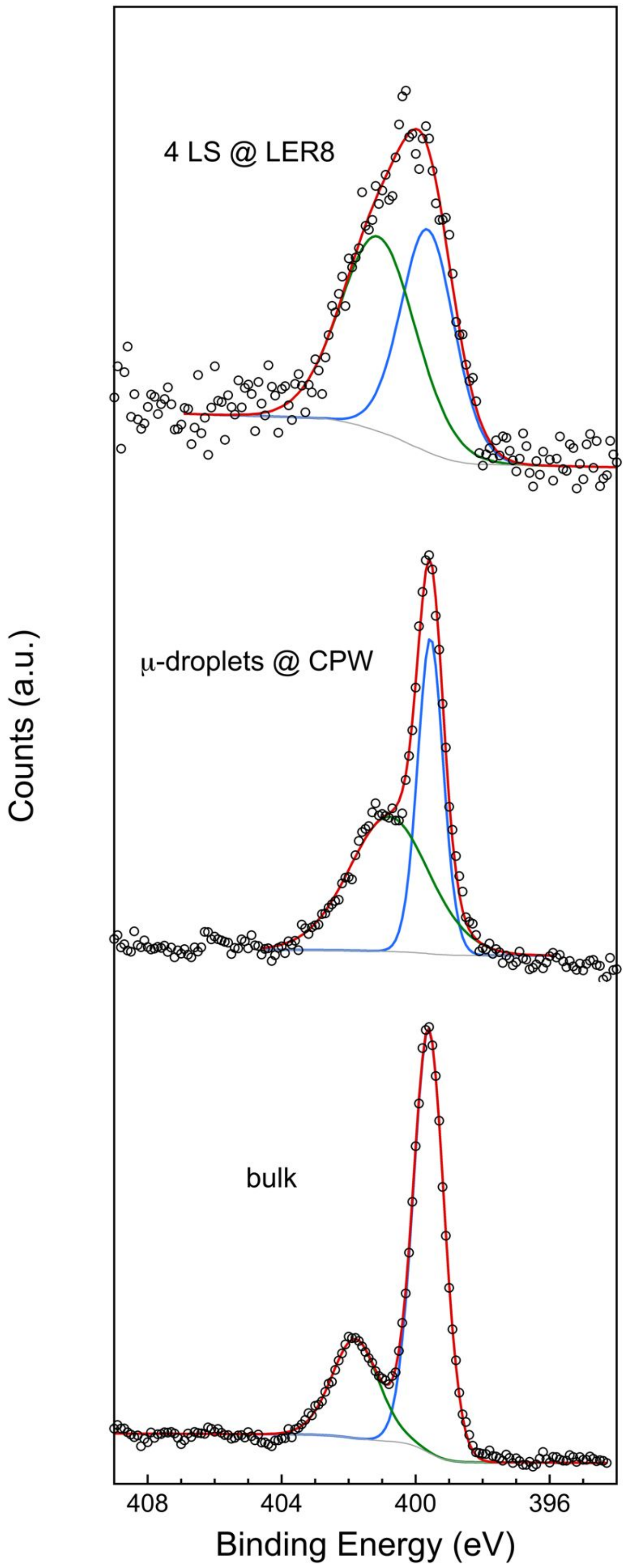


Figure S12. High resolution XPS N 1s spectra of the 4-layer LS deposit on LER8, compared to those previously reported for the bulk material and a deposit grown from μ-droplets on a CPW resonator.[2] Grey, blue, green and red solid lines are respectively the background (grey), components 1 and 2, and the envelope simulated spectra.

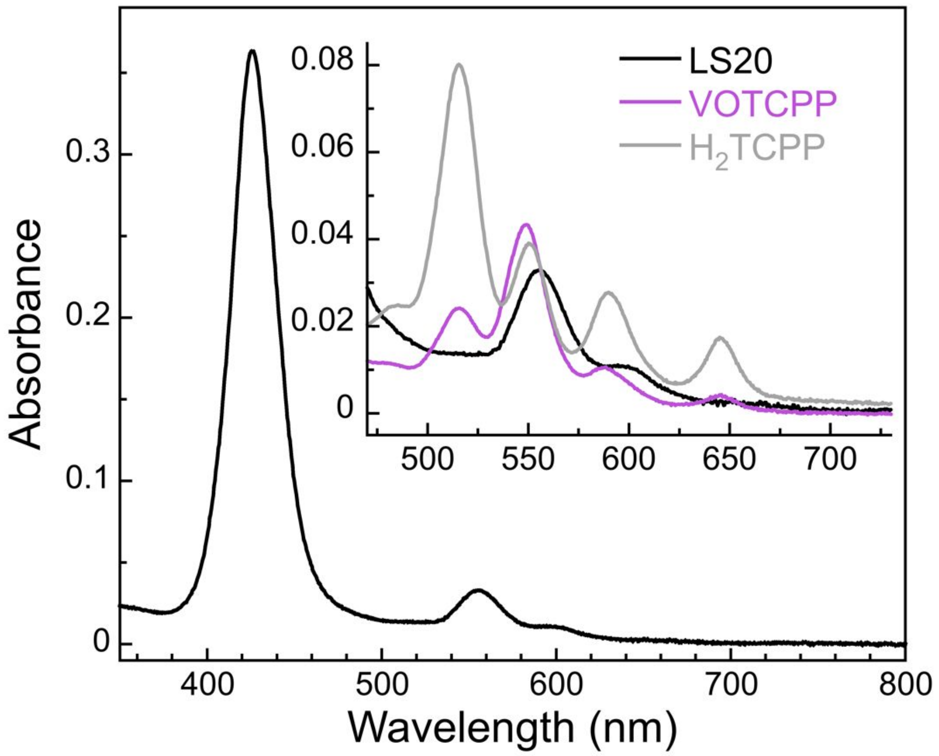


Figure S13. UV-Vis spectrum of a 20 LS film of [{VOTCPP}$Zn_2(H_2O)_2$] on quartz. The inset highlights the Q-bands area, comparing with the solution spectra of the free-base $H_2TCPP$ and [VOTCPP] in $CHCl_3$:MeOH 3:1, respectively 0.03 and 0.0368 mM.[2]

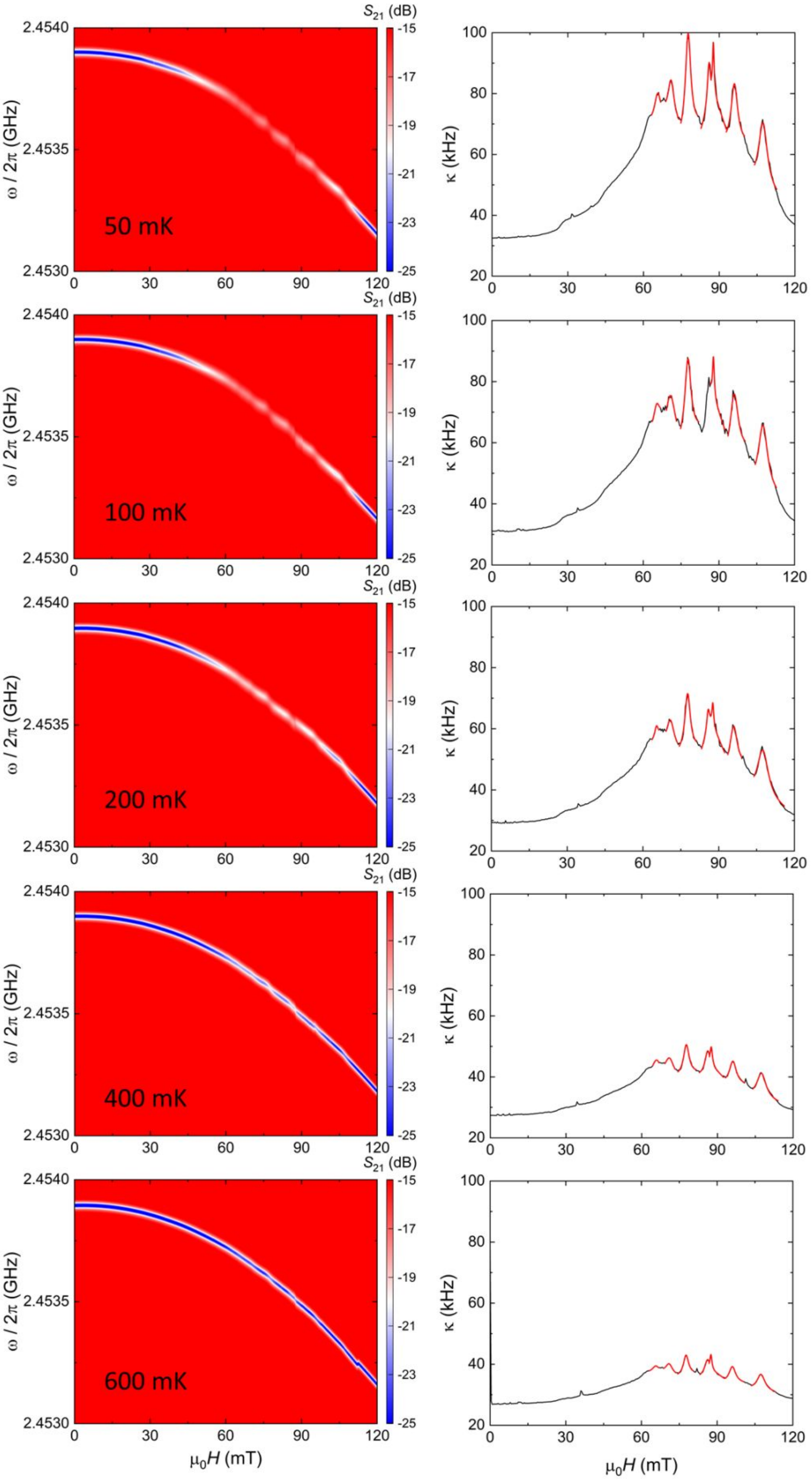


Figure S14. Color plot of the microwave transmission as a function of the applied magnetic field measured at the indicated temperatures near the bare resonance of LER5 covered with *ca.* $1.10\times10^{12}$ vanadyl spins (left) and the corresponding field dependence of the linewidth of the coupled spin-LER system (right). The actual number of spins on the inductor lines, *i.e.* those coupled to the LER, is *ca.* 4/25 the total, *i.e.* $1.76\times10^{11}$.

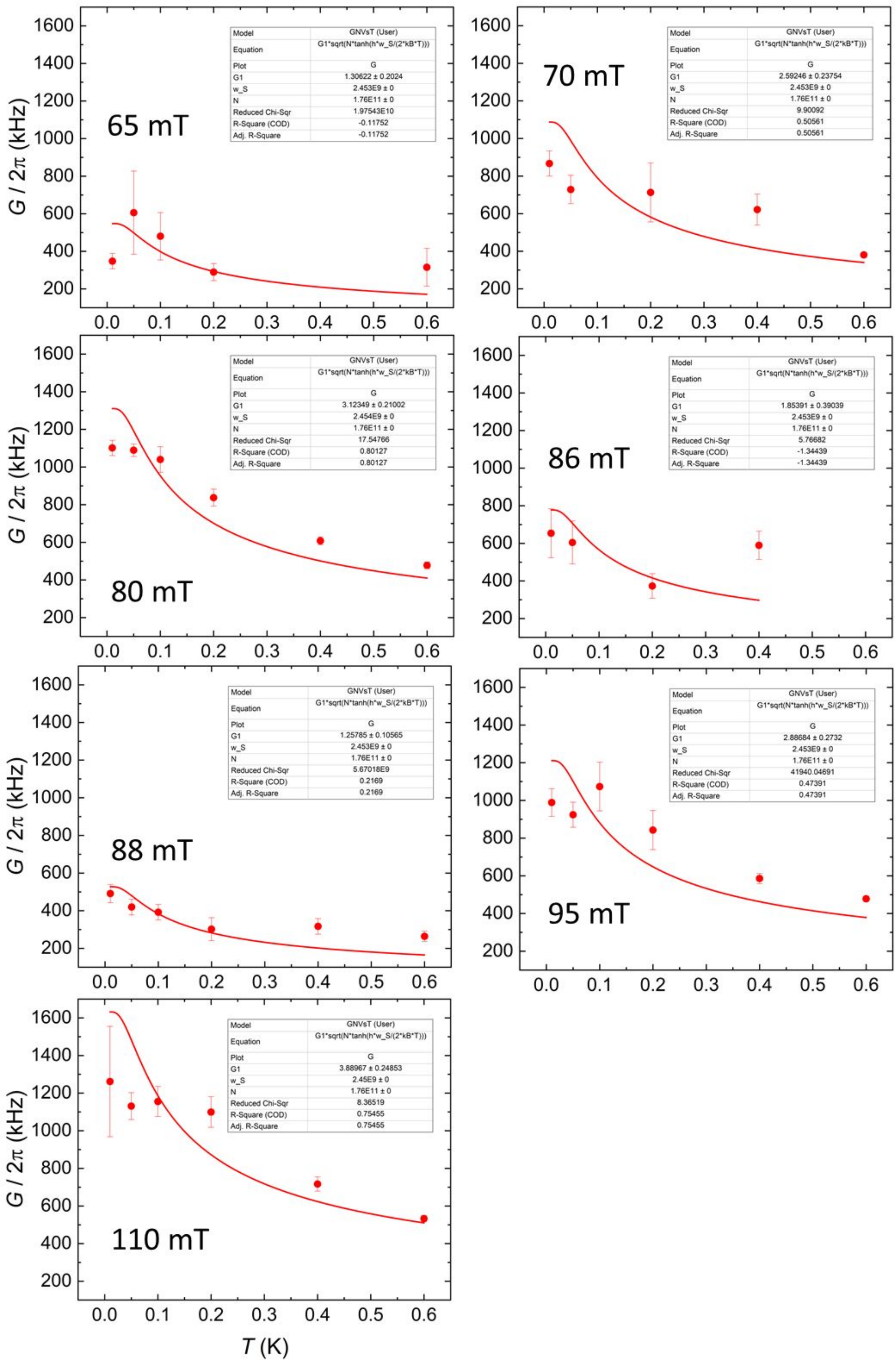


Figure S15. Temperature dependence of the collective spin-photon coupling $G_N/2\pi$ determined at the different maxima in the field dependence of the linewidth of LER5 covered with *ca.* $1.10\times10^{12}$ vanadyl spins. The actual number of spins on the inductor lines, *i.e.* those coupled to the LER, is *ca.* 4/25 the total, *i.e.* $1.76\times10^{11}$.

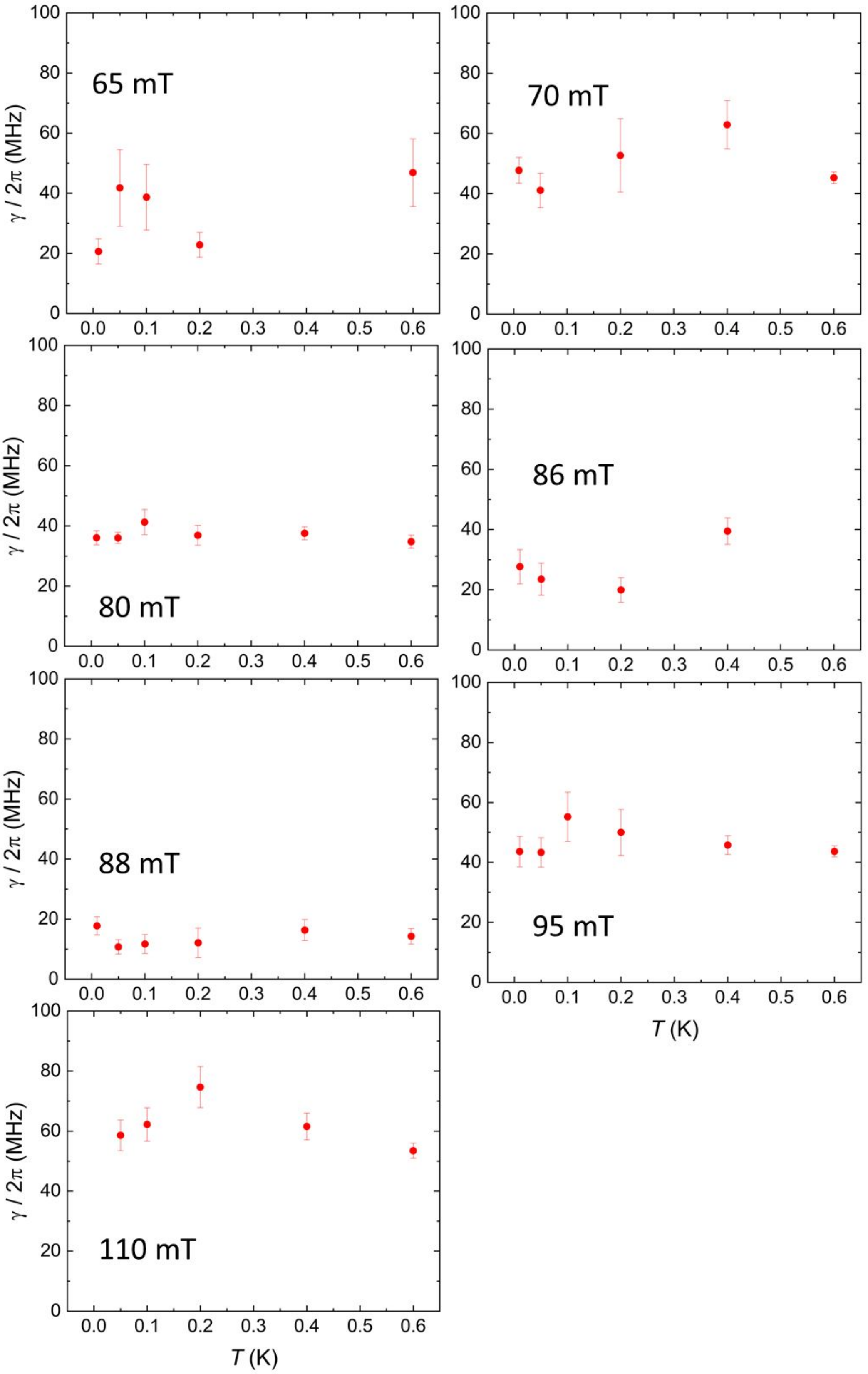


Figure S16. Temperature dependence of the line width of the different maxima in the field dependence of $\kappa$ of LER5 covered with *ca.* $1.10\times10^{12}$ vanadyl spins. The actual number of spins on the inductor lines, *i.e.* those coupled to the LER, is *ca.* 4/25 the total, *i.e.* $1.76\times10^{11}$.

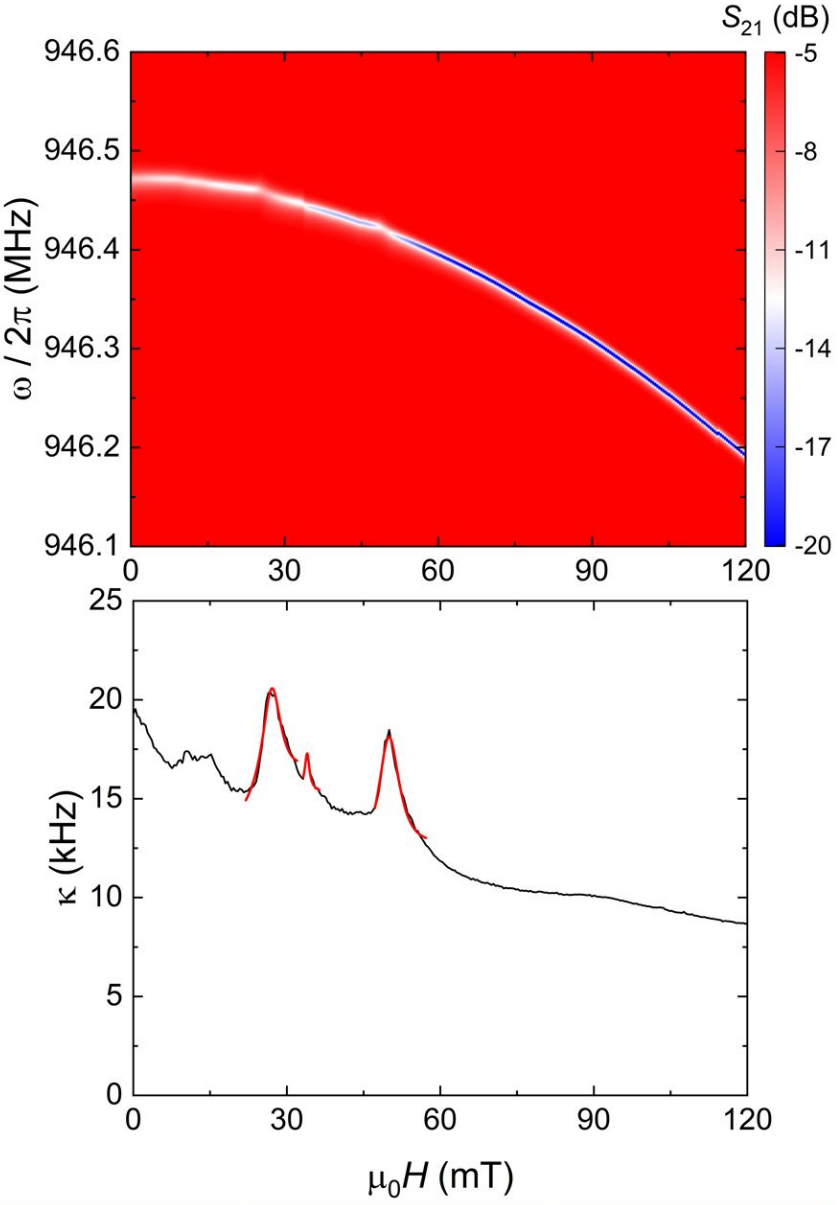


| $\mu_0 H$ (mT) | $G_N/2\pi$ (kHz) | $\gamma/2\pi$ (MHz) |
|---|---|---|
| 27 | $554 \pm 76$ | $55 \pm 9$ |
| 34 | $162 \pm 68$ | $15 \pm 2$ |
| 50 | $671 \pm 71$ | $71 \pm 8$ |

Figure S17. Color plot of the microwave transmission as a function of the applied magnetic field measured at 10 mK near the bare resonance (0.728 GHz) of LER7 covered with *ca.* $2.43\times10^{12}$ vanadyl spins (top) and the corresponding field dependence of the linewidth of the coupled spin-LER system (middle). Red lines are fits of each maxima to a Lorentzian line shape, providing the collective spin-photon coupling $G_N/2\pi$ and line width $\gamma$ given in the table (bottom). The actual number of spins on the inductor lines, *i.e.* those coupled to the LER, is *ca.* 4/25 the total, *i.e.* $3.89\times10^{11}$.

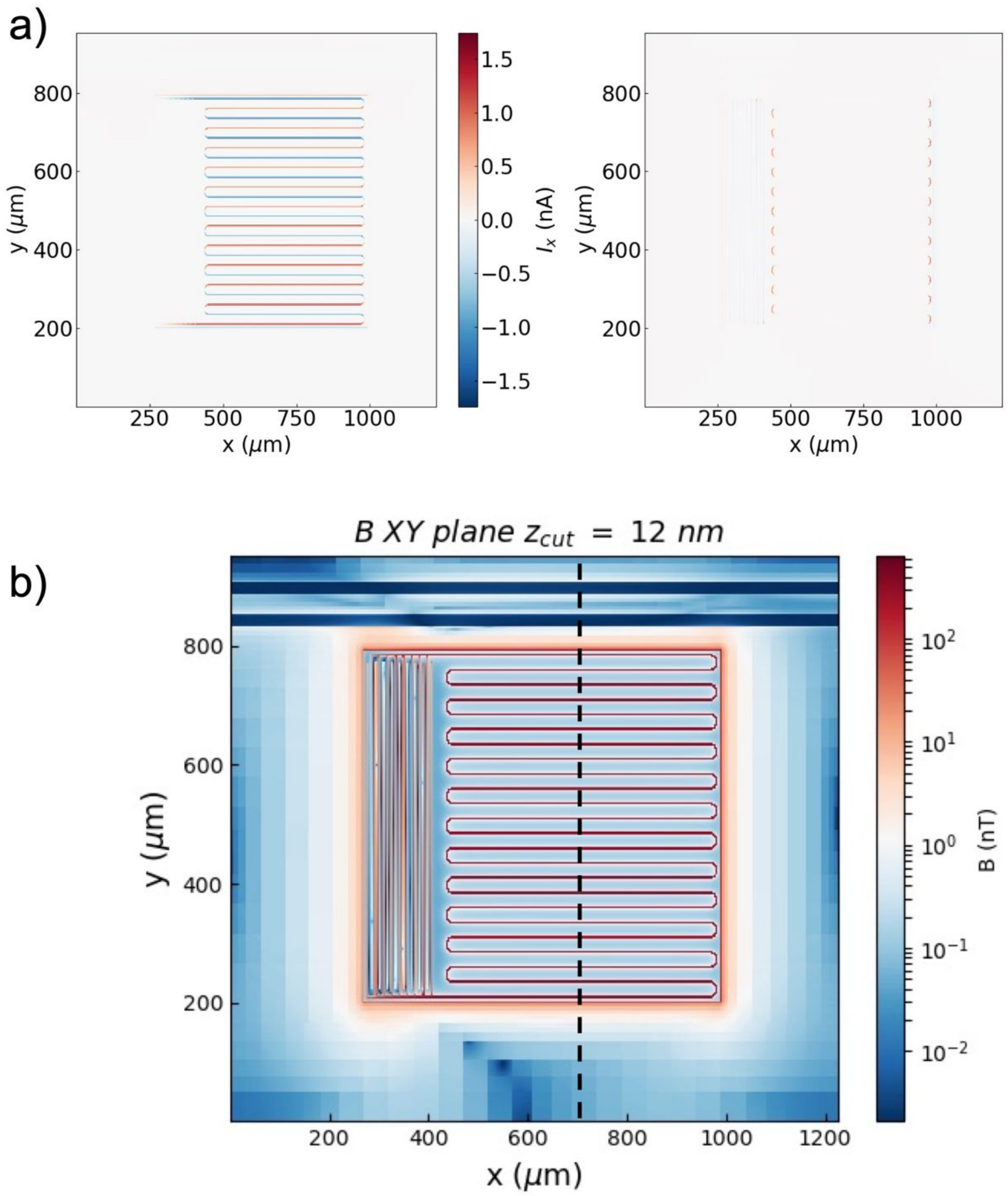


Figure S18. a) Sonnet simulation of the current in LER5 normalized to 1 photon. Calculations were made using 0.5 × 0.5 μm$^2$ cells. b) Map of the photon magnetic field at 12 nm off the surface of LER5. Simulations were made along z from 5 to 55 nm atop the surface, realizing 1 nm cuts. Shown is the cut at z = 12 nm, *i.e.* approximately in the middle of the 4-layer LS film, which has 20-24 nm height. A 1D cut at x = 700 μm, shown as a dashed line, gives values of *B* of 0.207 nT in the middle of the valleys between the inductor lines and 190.082 nT at the centre of the inductor lines. This results in a ratio of the coupling of spins in these two areas $g_{valleys}/g_{lines} \simeq 10^{-6}$, i.e. that only the spins on top of the inductor lines actually couple to the photons in the resonator. Considering the geometry of the inductor (4 μm wide lines, 21 μm valleys in between), the ratio of the number of spins in the two areas is $n_{valleys}/n_{lines} \simeq 21/4$. Then, $G_N \simeq \sqrt{n_{lines}g_{lines}^2 + n_{valleys}g_{valleys}^2} \simeq \sqrt{n_{lines}}g_{lines}$, with $n_{lines} \simeq (4/25)N_{total}$, $N_{total}$ being the estimated number of spins contained in the film covering the whole inductor area, in the case of LER5, 1.10×10$^{12}$.

[1] D. Nečas, P. Klapetek, Gwyddion: an open-source software for SPM data analysis, *Cent. Eur. J. Phys.*, **2012**, 10, 181–188
[2] A. Urtizberea, E. Natividad, P. J. Alonso, L. Pérez-Martínez, M. A. Andrés, I. Gascón, I. Gimeno, F. Luis, O. Roubeau, Vanadyl spin qubit 2D arrays and their integration on superconducting resonators, *Mater. Horiz.*, **2020**, 7, 885-897
[3] a) R. Makiura, S. Motoyama, Y. Umemura, H. Yamanaka, O. Sakata, H. Kitagawa, Surface nano-architecture of a metal–organic framework. *Nat. Mater.*, **2010**, 9, 565; S. Motoyama, R. Makiura, O. Sakata, H. Kitagawa, Highly Crystalline Nanofilm by Layering of Porphyrin Metal−Organic Framework Sheets. *J. Am. Chem. Soc.*, **2011**, 133, 5640
[4] K. A. Macor, R. S. Czernuszewicz, T. G. Spiro, Influence of porphyrin radical type on vanadium-oxygen double bond strength in vanadyl porphyrin cation radicals: implications for heme protein intermediates. *Inorg. Chem.*, **1990**, 29, 1996
[5] N. P. Kazmierczak, N. E. Lopez, K. M. Luedecke, R. Hadt, Determining the key vibrations for spin relaxation in ruffled Cu(ii) porphyrins via resonance Raman spectroscopy, *Chem. Sci.*, **2024**, 15, 2380
